# A Multicellular Network Mechanism for Temperature-Robust Food Sensing

**DOI:** 10.1016/j.celrep.2020.108521

**Published:** 2020-12-22

**Authors:** Dhaval S. Patel, Giovanni Diana, Eugeni V. Entchev, Mei Zhan, Hang Lu, QueeLim Ch’ng

**Affiliations:** 1 Centre for Developmental Neurobiology, King’s College London, London SE1 1UL, UK; 2 Interdisciplinary Bioengineering Graduate Program, Georgia Institute of Technology, Atlanta, GA 30332-0100, USA; 3 Wallace H. Coulter Department of Biomedical Engineering, Georgia Institute of Technology, Atlanta, GA 30332-0100, USA; 4 School of Chemical and Biomolecular Engineering, Georgia Institute of Technology, Atlanta, GA 30332-0100, USA

**Keywords:** robustness, gene networks, network plasticity, nutrient-sensing, lifespan, *C. elegans*, temperature, gene-environment interaction, serotonin, TGF-β

## Abstract

Responsiveness to external cues is a hallmark of biological systems. In complex environments, it is crucial for organisms to remain responsive to specific inputs even as other internal or external factors fluctuate. Here, we show how the nematode *Caenorhabditis elegans* can discriminate between different food levels to modulate its lifespan despite temperature perturbations. This end-to-end robustness from environment to physiology is mediated by food-sensing neurons that communicate via transforming growth factor β (TGF-β) and serotonin signals to form a multicellular gene network. Specific regulations in this network change sign with temperature to maintain similar food responsiveness in the lifespan output. In contrast to robustness of stereotyped outputs, our findings uncover a more complex robustness process involving the higher order function of discrimination in food responsiveness. This process involves rewiring a multicellular network to compensate for temperature and provides a basis for understanding gene-environment interactions. Together, our findings unveil sensory computations that integrate environmental cues to govern physiology.

## Highlights

• *C. elegans*’ ability to modulate lifespan in response to food is robust to temperature
• Robustness requires TGF-β and serotonin signaling in a neuronal network
• Specific regulations in the neuronal network change sign with temperature
• Temperature-dependent regulations compensate for temperature

## Graphical Abstract

**Figure:**
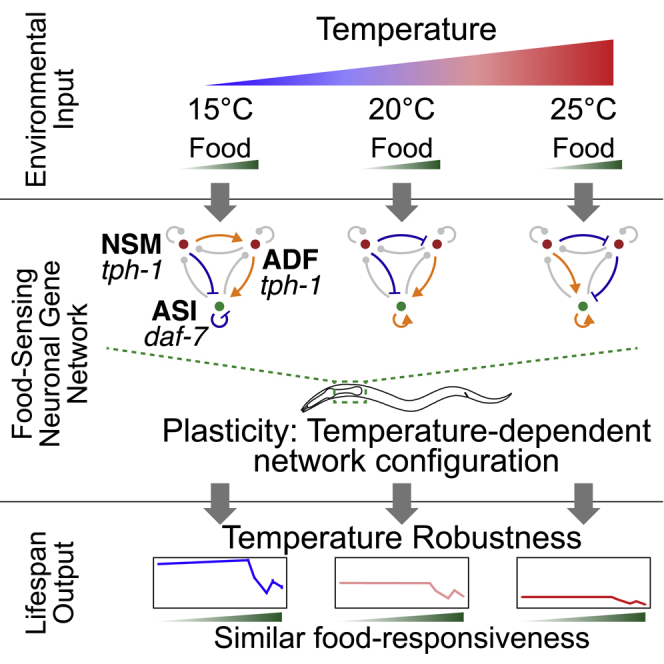

## Teaser

Both food and temperature impact lifespan, but how do they interact? Patel et al. report that the effect of dietary restriction on lifespan is robust to temperature. This ability is sustained by temperature-dependent plasticity in a food-sensory network. Such compensatory mechanisms maintain flexibility and responsiveness despite external perturbations.

## Introduction

Robustness is the ability of a system to maintain its performance under perturbation (Félix and Barkoulas, 2015; Hiesinger and Hassan, 2018; Kitano, 2004). Robustness is fundamental to biological systems, enabling organisms to thrive despite fluctuations in internal processes or external environments. Mechanisms for robustness exist in many precise and stereotyped processes, such as development (Félix and Barkoulas, 2015; Hiesinger and Hassan, 2018), circadian rhythms (Mohawk et al., 2012), and rhythmic neural activity (Marder et al., 2015), to produce invariant outputs despite internal variability in signaling activities or external perturbations in environmental conditions.

Unlike these stereotyped processes, much less is known about robustness in responsive processes in metazoans, where the ability to respond to one environmental cue is maintained despite fluctuations in a second factor that impact the same process. This alternative form of robustness is crucial in complex natural environments where multiple factors can fluctuate independently. A rigorous understanding of robustness necessitates an explicit definition of the biological parameter that is robust, and the perturbation to which it is robust (Felix and Barkoulas, 2015). Here, we investigate how food sensing is robust to temperature in the nematode *C. elegans*. This ability ensures that *C. elegans* can modulate its lifespan appropriately in response to food abundance over a broad range of temperatures.

Food- and nutrient-sensing pathways link food abundance to diverse physiological outputs in many species (Alcedo et al., 2010; Bishop and Guarente, 2007; Fontana and Partridge, 2015; Greer and Brunet, 2009; Kapahi et al., 2017; Lemieux and Ashrafi, 2015; Riera and Dillin, 2016; Tao et al., 2016; Templeman and Murphy, 2018). Transforming growth factor β (TGF-β) and serotonin comprise two conserved signaling pathways that act in different food-sensing neurons to regulate development, metabolism, longevity, and other processes (Ashrafi, 2007; Brown and Schneyer, 2010; Chang et al., 2006; Oury and Karsenty, 2011; Ren et al., 1996; Shaw et al., 2007; Sze et al., 2000; Zhang et al., 2005). We have previously shown that in *C. elegans*, disrupting both of these pathways by gene deletions of *daf-7* (encodes TGF-β [Ren et al., 1996]) or *tph-1* (encodes tryptophan hydroxylase, the rate-limiting enzyme in serotonin synthesis [Sze et al., 2000]) impairs both food-dependent increases and decreases in lifespan (Entchev et al., 2015), indicating that *tph-1* and *daf-7* possess the unique ability to bidirectionally modulate the food responsiveness of lifespan. Furthermore, mutations in both *tph-1* and *daf-7* ablate ∼80% of the ability to modulate lifespan in response to food (Diana et al., 2017), indicating that these pathways constitute major routes for transmitting food-related information in this process.

Sensory neurons are the primary detectors of food within the nervous system that transmit nutritional information to different tissues, ensuring that the organism can adjust its physiology based on food availability. Both *tph-1* and *daf-7* are expressed in and act from a set of food-responsive sensory neurons to modulate lifespan: *tph-1* in NSM and ADF sensory neurons and *daf-7* in ASI sensory neurons (Cunningham et al., 2012; Entchev et al., 2015; Fletcher and Kim, 2017; Gallagher et al., 2013; Ren et al., 1996; Rhoades et al., 2019; Schackwitz et al., 1996; Sze et al., 2000; You et al., 2008; Zaslaver et al., 2015). In these food-sensing neurons, the expression levels of *tph-1* and *daf-7* are regulated by food levels to affect lifespan, thereby providing an internal representation of food abundance that modulates a critical functional output (Entchev et al., 2015; Fletcher and Kim, 2017). Cross- and self-regulation among these genes in their respective cells indicate that they act in a gene network distributed over multiple cells (Chang et al., 2006; Entchev et al., 2015; Sze et al., 2000).

Temperature also affects lifespan in *C. elegans* and other species (Conti, 2008; Lee and Kenyon, 2009; Riera and Dillin, 2016; Stroustrup et al., 2016; Xiao et al., 2013). There is long-standing interest in how complex environmental conditions interact to impact lifespan, but the relationship between food and temperature during lifespan modulation has remained unclear. By analyzing lifespan across a systematic combination of food and temperature regimens, we find that the ability to modulate lifespan in response to food availability is robust to temperature. Through quantitative analysis of the multicellular gene network involving *tph-1* and *daf-7*, we discover that this food-sensing network changes the sign of specific regulatory connections in a temperature-dependent way to maintain this form of robustness.

## Results

### Food and Temperature Interact to Modulate *C. elegans* Lifespan

**Figure 1:**
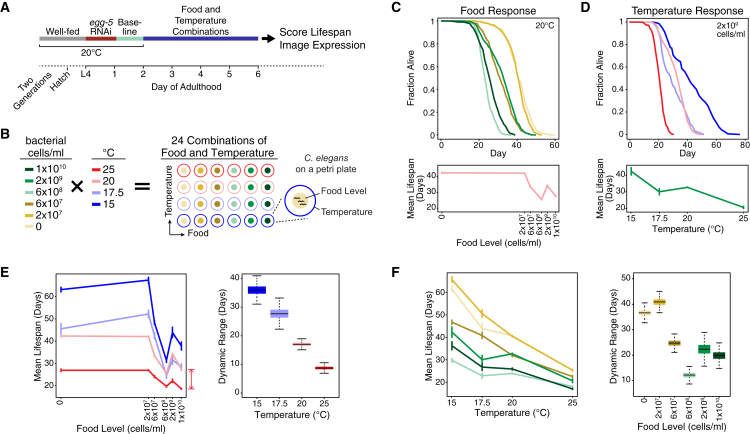
Food and Temperature Exert Combinatorial Effects on *C. elegans* Lifespan (A) Food and temperature manipulations. Animals were shifted to specific food and temperature combinations after which their lifespan or gene expression levels were measured. (B) Food and temperature combinations tested. (C) Effect of food levels on wild-type lifespan at 20°C. Top: Kaplan-Meier survival curves for each food level. Bottom: mean lifespan across food levels (Table S1). (D) Effect of temperature on wild-type lifespan at 2 × 10^9^ bacterial cells/mL. Top: Kaplan-Meier survival curves for each temperature. Bottom: mean lifespan across temperature (Table S1). (E) Effect of temperature on food responsiveness of lifespan. Left: mean lifespan across food levels at different temperatures, with the definition of the dynamic range at 25°C illustrated by the red bar. Right: distributions of the dynamic range of food responsiveness at each temperature shown as boxplots, where the filled box indicates the 25^th^–75^th^ percentile of the distribution (the inter-quartile range), the line within the box denotes the median, and the span of the whiskers reflects the range of the distribution (see Table S2 for quantification of overlap). (F) Effect of food on temperature responsiveness of lifespan. Left: mean lifespan across temperature at different food levels. Right: distributions of the dynamic range of temperature responsiveness indicated by boxplots as described in (E). For wild type, n = 112–525 for the 24 food and temperature conditions tested; please see Table S1 for detailed sample sizes. Bayesian estimates are shown for all mean lifespans and dynamic ranges (see STAR Methods and Table S1). Error bars in all line plots in (C)–(F) indicate standard deviations.

The effect of food and temperature on lifespan can be observed when *C. elegans* are shifted to specific food and temperature levels during their reproductive period on day 2 of adulthood (Figures 1A and 1B; see STAR Methods) (Entchev et al., 2015; Patel et al., 2017). In our previous study, we examined the lifespan response of wild-type animals across 19 different food concentrations and found a multifaceted relationship between food availability and longevity (Entchev et al., 2015). The complexity of wild-type lifespan as a function of food abundance is captured by examining six specific food concentrations that represent key points in the food response (Entchev et al., 2015). At 20°C, decreasing bacterial food concentration from *ad libitum* (1 × 10^10^ bacterial cells/mL) to starvation (0 bacterial cells/mL) leads to local maxima and minima in lifespan, which plateau to a maximum at the lowest food levels (Figure 1C; Table S1). At a baseline food level (2 × 10^9^ bacterial cells/mL), lifespan is extended by decreasing temperature from 25°C to 15°C (Figure 1D; Table S1). These temperatures constitute the non-stressful range where *C. elegans* is viable (Félix and Braendle, 2010). Because *C. elegans* adopts a boom-and-bust lifestyle in temperate climates (Schulenburg and Félix, 2017), these food and temperature ranges are consistent with fluctuations seen in its natural environment.

To determine how these environmental factors interact, we measured lifespan under 24 combinations of food and temperature (Figure 1B). We then stratified the results by food or temperature to reveal the effect of temperature on food responsiveness and vice versa. At temperatures from 15°C to 25°C, the basic shape of the food-lifespan relationship remained similar even as increasing temperature reduced lifespan (Figure 1E, left). The dynamic range of this food response can be measured by the difference between the highest and lowest mean lifespans across food levels. Increased temperature also significantly compressed the dynamic range of the food response, as shown by their completely non-overlapping distributions at 15°C and 25°C (Figure 1E, right; Table S2; see STAR Methods for a detailed description of the statistical interpretations from boxplots used throughout the figures). At food levels from starvation to *ad libitum*, increasing temperature generally reduced lifespan (Figure 1F, left). However, the dynamic range of the temperature response varied considerably with food, as shown by several non-overlapping distributions (e.g., from no food to 6 × 10^7^ and 6 × 10^8^ bacterial cells/mL), and was highest at the lowest food levels (Figure 1F, right).

### Food Discrimination Is Robust to Temperature

**Figure 2:**
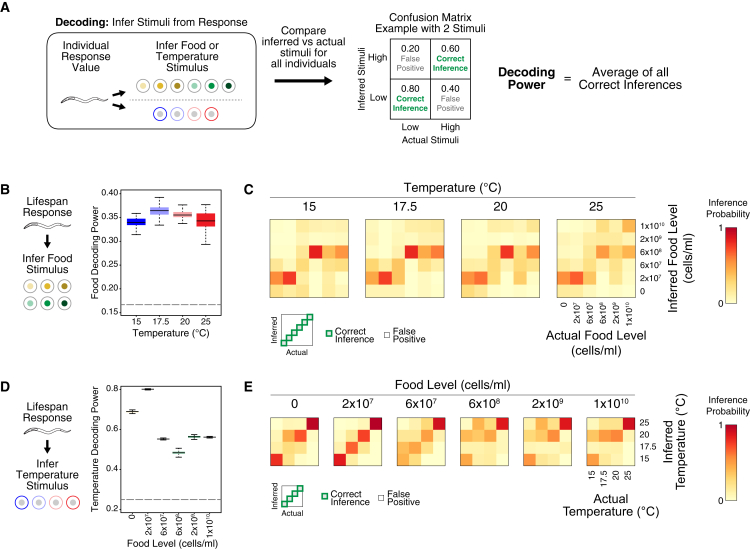
Food Responsiveness Is Specifically Robust to Temperature (A) Scheme for decoding analysis to quantify discrimination, whereby stimuli inferred based on the responses are compared with the actual stimuli (see text and STAR Methods). Examples of inferred frequencies in the confusion matrix are provided to illustrate the analysis output. A high frequency of correct inference indicates good discrimination. (B) Food decoding power at different temperatures based on lifespan responses at each temperature (see Table S3 for quantification of overlap). (C) Confusion matrices corresponding to food decoding for each temperature in wild-type animals. Each matrix indicates how frequently a food level was inferred, given the actual food stimulus. As indicated by the legend (bottom right), the diagonals represent the frequency of correct inferences, where the actual and inferred food levels are identical, whereas incorrect inferences are indicated by squares outside the diagonal. These confusion matrices reveal that based on the lifespan responses, some food levels are well discriminated, whereas other food levels are not. At all temperatures, wild-type animals can easily discriminate 2 × 10^7^ and 6 × 10^8^ bacterial cells/mL from other food levels but tend to mistake no food for 2 × 10^7^ bacterial cells/mL. This inference pattern is stable in wild type across temperature, only changing slightly at 25°C, reflecting the robustness of food sensing with temperature. The color bar on the right indicates the inference probability. (D) Temperature decoding power at different food levels based on lifespan responses at each food level. (E) Confusion matrices for temperature decoding at each food level. For wild type, n = 112–525 for the 24 food and temperature conditions tested; please see Table S1 for detailed sample sizes. In (B) and (D), Bayesian distributions are depicted by boxplots as described for Figure 1E, and dotted lines indicate decoding power from random chance alone.

This ability to produce different lifespans under different environmental conditions implies that the physiological systems in *C. elegans* can discriminate among them. We used decoding analysis (Dayan and Abbott, 2005) to quantify how well environmental conditions can be discriminated based on the corresponding distributions of lifespan or gene expression. This method has been successfully applied to gene expression, biochemical activities, and physiological responses (Entchev et al., 2015; Granados et al., 2018) and accommodates noisy responses and non-linear stimuli-response relationships. Using this approach (Figure 2A; see STAR Methods), we first infer the most likely stimulus for a given response, using prior knowledge of the response distributions under different stimuli. Next, we compared the inferred versus actual stimuli to determine the frequency of correct and incorrect inferences under each stimulus, generating a confusion matrix of inference patterns depicting how well different stimuli are distinguishable from each other and the frequency of erroneous inferences. Finally, we use the average frequency of correct inferences, which we term decoding power, to summarize the discriminatory performance. Higher decoding power is associated with a stronger coupling between stimuli and response, indicating better discrimination and hence superior performance.

For each temperature, we used decoding analysis on data from that temperature to quantify discrimination between food levels that produces different lifespans. Remarkably, food decoding power in wild-type animals remained constant from 15°C to 25°C (Figure 2B; Table S3), despite the large changes in the absolute lifespan values (Figure 1E). Some food levels were discriminated well and others poorly, indicating that *C. elegans* is tuned to specific food levels; for example, at 15°C (Figure 2C, leftmost matrix), animals could easily identify food levels consisting of 2 × 10^7^ and 6 × 10^8^ bacterial cells/mL (76% and 83% correct inference, respectively), but not other food levels (4%–26% correct inference). This heterogeneous and specific inference pattern underlying the average decoding power was also preserved across a 10°C range (Figure 2C). Thus, discrimination between food levels is robust to temperature, as performance remained similar at all tested temperatures. The robustness of food discrimination to temperature is specific: although discrimination between temperatures is relatively strong, this performance fluctuates considerably as food levels are changed (Figures 2D and 2E), as indicated by non-overlapping temperature decoding power at different food levels. Thus, temperature discrimination is not robust to food. This form of robustness represents an unexpected interaction between food and temperature during lifespan modulation; it is biologically significant because it spans environmental input to physiological output.

### Serotonin and TGF-β Signals Are Required for Temperature-Robust Food Responsiveness

**Figure 3:**
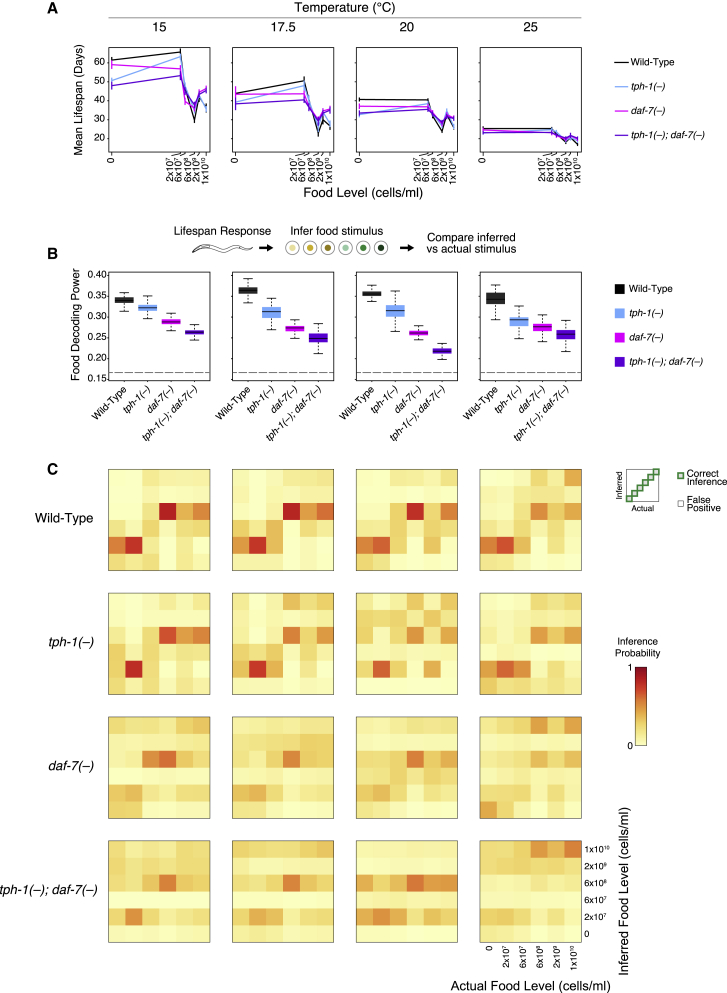
*tph-1* and *daf-7* Are Required for Temperature-Robust Food Responsiveness in Lifespan (A) Mean lifespan for each genotype as a function of food levels under different temperatures. Loss of *tph-1* and *daf-7* attenuate food responsiveness bidirectionally at all temperatures. Bayesian estimates are shown for all mean lifespans (see STAR Methods and Table S1). Error bars in all line plots indicate standard deviations. (B) Top: summary of the food decoding process. Bottom: food discrimination based on the lifespan response is impaired in *tph-1(−)* and *daf-7(−)* at all temperatures, as measured by food decoding power. Bayesian distributions are depicted by boxplots as described for Figure 1E. Quantification of the overlap between distributions can be found in Table S3. Dotted lines indicate decoding power from random chance alone. (C) A grid of confusion matrices corresponding to food decoding for each temperature (grid column) and genotype (grid row). In contrast to the relatively stable inference patterns in wild type, the inference patterns in the *tph-1(−)* and *daf-7(−)* mutants are unstable and vary dramatically with temperature. See also related Figures S1 and S2. Range of sample sizes for the 24 food and temperature conditions tested are as follows: wild type n = 112–525, *tph-1(−)* n = 84–209, *daf-7(−)* n = 142–214, *tph-1(−);daf-7(−)* n = 130–213 (please see Table S1 for detailed sample sizes).

To understand how robustness arises, we assessed the role of food sensing pathways. Since serotonin and TGF-β represent a major link between food and lifespan (Diana et al., 2017; Entchev et al., 2015; Fletcher and Kim, 2017), we assessed the contributions of *tph-1* and *daf-7* to discrimination of food levels at different temperatures (Figure 3). At all temperatures, loss of *tph-1* and *daf-7* attenuated both increases and decreases in lifespan due to food, with the most severe attenuation observed in the double mutant (Figure 3A; Table S1). This attenuation was most obvious under starvation and at 6 × 10^8^ bacterial cells/mL, where the respective food-dependent lifespan extension and reduction occurred with smaller magnitudes in the single and double mutants compared with wild type (Figure 3A).

Decoding analysis of the lifespan responses revealed that at all temperatures tested, food decoding power was reduced in both *tph-1(−)* and *daf-7(−)* animals, with the double mutant displaying the most severe effects (Figure 3B). The magnitudes of these effects are large, as shown by the completely non-overlapping food decoding power between the wild type and the double mutant (Figure 3B; Table S3). These reductions in food decoding power were observed even when the analysis was repeated with each of the individual food levels left out (Figure S1), indicating that we had tested a sufficiently informative set of food levels. While the pattern of correct and incorrect inferences in wild type were largely stable to temperature, these patterns became more temperature sensitive in *tph-1(−)* and *daf-7(−)* single and double mutants (Figure 3C), implying that unlike wild type, the mutants were attuned to different food levels at different temperatures. Thus, this effect on food decoding power is not a trivial consequence of *tph-1(−)* and *daf-7(−)* mutants being unable to sense food. Instead, they indicate that *tph-1* and *daf-7* contribute to the robustness of food discrimination to temperature perturbations.

The ability to discriminate between temperatures is highly sensitive to the food level (Figure 2D), and loss of *tph-1* and/or *daf-7* did not produce any consistent effect on temperature discrimination (Figure S2). Mutations in these genes could either enhance or reduce the effects of temperature on lifespan, depending on the food level (Figure S2A), leading to corresponding increases or decreases in temperature decoding power as a function of food (Figures S2B and S2C). Taken together, we conclude that the effects of *tph-1* and *daf-7* on robustness are specific to one functional parameter (food level discrimination) under one perturbation (temperature).

### Increased Food Responsiveness in the *tph-1*/*daf-7* Network Compensates for Temperature

**Figure 4:**
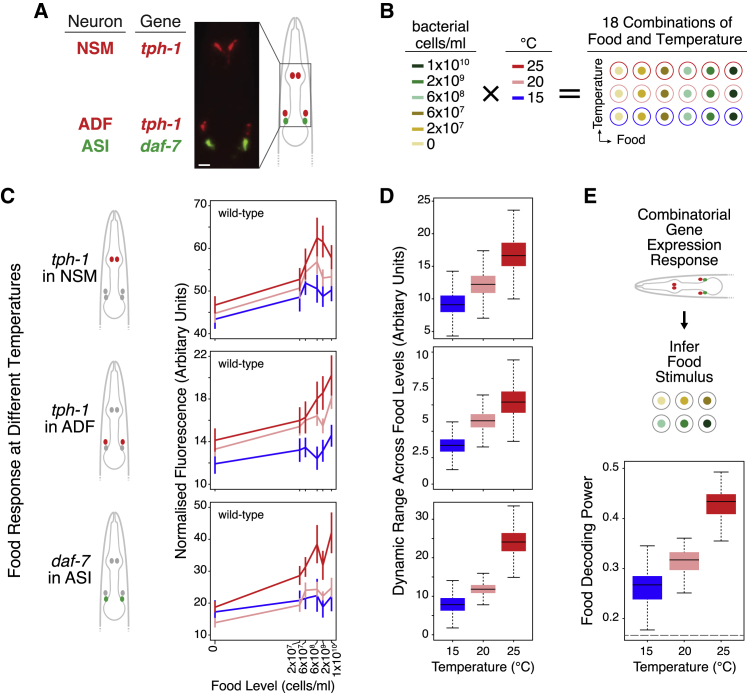
Food- and Temperature-Responsive Gene Expression *tph-1* and *daf-7* (A) Transcriptional reporters showing *tph-1* expression in NSM and ADF neurons and *daf-7* expression in ASI neurons. Scale bar indicates 10 μm. (B) Eighteen food and temperature combinations tested. (C) Wild-type expression of *tph-1* in NSM and ADF and *daf-7* in ASI as a function of food. Each line corresponds to a different temperature (see Table S4). Error bars denote 90% confidence intervals. Diagrams on the left denote gene and cell. (D) Dynamic range of food responsiveness in expression of *tph-1* in NSM and ADF and *daf-7* in ASI at different temperatures (see Table S5). (E) Wild-type food decoding power at different temperatures based on the combinatorial expression of *tph-1* and *daf-7* in these three cells (see Table S6). Distribution of the values in (D) and (E) are depicted by boxplots as described in Figure 1E. For wild type, n = 405–2,958 for the 18 food and temperature conditions tested; please see Table S4 for detailed sample sizes. Bayesian estimates are shown for all mean expression values and dynamic ranges (see STAR Methods and Table S4). Dotted line in (E) indicates decoding power from random chance alone. The data in (C)–(E) are plotted as a function of temperature in Figure S3.

Robustness could be achieved in two general ways: either by resisting the perturbation and remaining unchanged or by adapting and compensating. To distinguish between these possibilities, we characterized the wild-type gene expression of *tph-1* in NSM and ADF neurons as well as *daf-7* in ASI neurons (Figure 4A) under a systematic combination of food levels and temperatures (Figure 4B). We used quantitative high-throughput imaging to measure expression of validated single-copy transcriptional reporters for *tph-1* and *daf-7* at the single-cell level in their respective neurons (Chung et al., 2008; Entchev et al., 2015; Zhan et al., 2015).

In wild-type animals, expression of *tph-1* in NSM and ADF or of *daf-7* in ASI shows distinct response profiles as a function of food based on these reporters, reflecting their food-encoding properties (Entchev et al., 2015) (Figures 4C and S3–S7; Table S4). While these food response profiles largely retained their shape from 15°C to 25°C, expression levels generally increased with higher temperatures (Figures 4C and S3). Additionally, the dynamic range of gene expression for both genes in all three sets of cells also increased with temperature (especially for *daf-7* expression in ASI, where the dynamic ranges at 15°C and 25°C have almost no overlap; see Table S5), indicating that these gene activities are more responsive to food levels (Figure 4D).

To quantify discrimination at the level of gene expression, we used decoding analysis. Having simultaneously imaged all three cells in each animal, we could infer stimuli based on the combinatorial expression values of *tph-1* and *daf-7* in NSM, ADF, and ASI (Figures 4E and S7). This analysis revealed that the food decoding power of this combinatorial gene expression increased with temperature (Figure 4E), to the extent that the food decoding power at 15°C and at 25°C form non-overlapping distributions (see Table S6). In other words, this network of cells could distinguish between food levels better as temperature was increased, consistent with the increased dynamic range of their food responses (Figure 4D).

These results suggest that robustness to temperature is achieved by changes in the underlying multicellular gene network, whereby the network adapts to compensate for temperature rather than remain unchanged. As temperature increases from 15°C to 25°C, the overall shape of the lifespan response to food availability remains similar, indicating robust food sensing; however, its dynamic range becomes compressed (Figure 1E) and the magnitudes of the effects of *tph-1* and *daf-7* on lifespan are reduced (Figure 3A). Maintaining a similar level of food responsiveness in the lifespan output requires a mechanism that compensates for this reduction in the effects of *tph-1* and *daf-7* activity on lifespan with increasing temperature. Increasing the dynamic range of food-responsive *tph-1* and *daf-7* expression (Figure 4D) heightens the food-dependent difference in gene activity within this network to compensate, at least in part, for the reduced impact of these genes on lifespan.

The increased decoding power from the combinatorial gene expression revealed that the discriminatory ability of the multicellular gene network is plastic and temperature dependent. This finding also raised the question of how the performance of such a higher order function as discrimination, which involves mapping multiple stimuli to corresponding responses, could be modulated.

### Architecture of the *tph-1*/*daf-7* Network Is Food and Temperature Dependent

**Figure 5:**
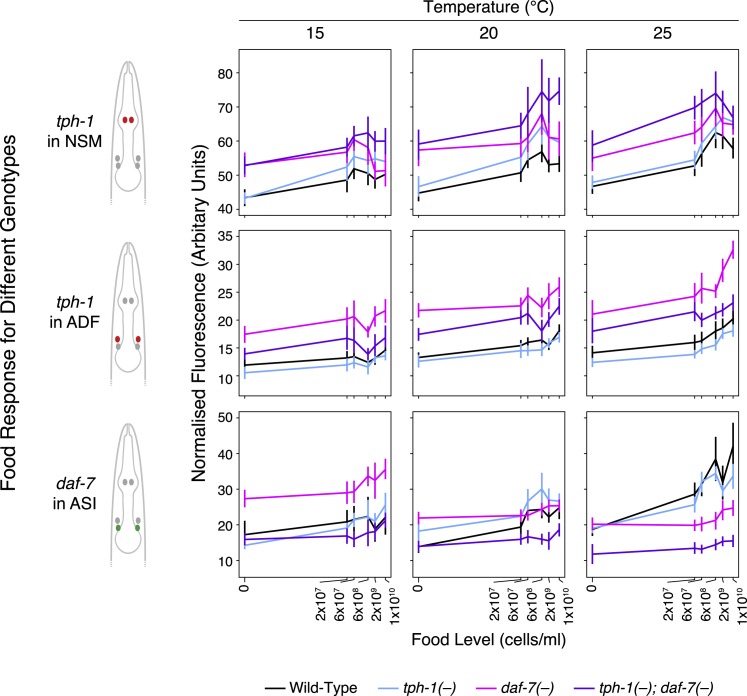
Cross- and Self-Regulation in the *tph-1*/*daf-7* Network Is Context Dependent Expression of *tph-1* in NSM and ADF and *daf-7* in ASI as a function of food at different temperatures in single and double mutants of *tph-1* and *daf-7*. Range of sample sizes for the 18 food and temperature conditions tested are as follows: wild type n = 405–2,958, *tph-1(−)* n = 70–313, *daf-7(−)* n = 90–191, *tph-1(−);daf-7(−)* n = 57–145 (please see Table S4 for detailed sample sizes). Bayesian estimates are shown for all mean expression values (see STAR Methods and Table S4). Error bars indicate 90% confidence intervals. Representative images are shown in Figure S4. To visualize this expression data as a function of temperature at different food levels, see Figure S5. See also related Figures S6 and S7.

We previously identified modulatory regulation among *tph-1* and *daf-7* at 20°C using transcriptional reporters to measure *tph-1* and *daf-7* expression levels in NSM, ADF, and ASI in single and double mutants of these genes at different food levels (Diana et al., 2017; Entchev et al., 2015). To understand how these interactions are affected by temperature, we analyzed the effects of *tph-1* and *daf-7* mutations on the expression of these reporters in their respective cells under more extensive food and temperature combinations (Figures 5 and S4–S7; Table S4). These results revealed how regulations among *tph-1* and *daf-7* change as a function of food and temperature. First, mutations in *tph-1* and *daf-7* could affect expression of *tph-1* and *daf-7* in all three cells, indicating that *tph-1* and *daf-7* act in a highly interconnected network with extensive cross- and self-regulation. Second, *tph-1* and *daf-7* act separately but interact to set gene expression levels. In all three cells, the expression phenotypes of *tph-1(−);daf-7(−)* double mutants largely differed from each of the single mutants (Figures 5 and S5). Furthermore, comparisons between *daf-7(−)* single mutants and *tph-1(−);daf-7(−)* double mutants show that *tph-1(−)* mutations have a more prominent effect in the *daf-7(−)* background (Figures 5 and S5). Third, gene-environment interactions were extensive, as food and temperature modified the effects of *tph-1(−)* and *daf-7(−)* mutations. Using *daf-7* expression in ASI as an example (Figures 5 and S5, bottom rows), loss of both *tph-1* and *daf-7* had a greater effect on *daf-7* expression in ASI at higher food levels. Also, loss of *daf-7* increased *daf-7* expression in ASI at 15°C but decreased it at 25°C, indicating a temperature dependence in *daf-7* self-regulation. Such temperature-dependent phenotypes suggest that the network configuration changes with temperature. Because *tph-1* and *daf-7* mediate robustness to temperature (Figures 2 and 3), our results suggest that these genes adopt different network configurations at different temperatures to produce similar food discrimination.

### Delineating Temperature-Dependent Connections in the *tph-1*/*daf-7* Network

**Figure 6:**
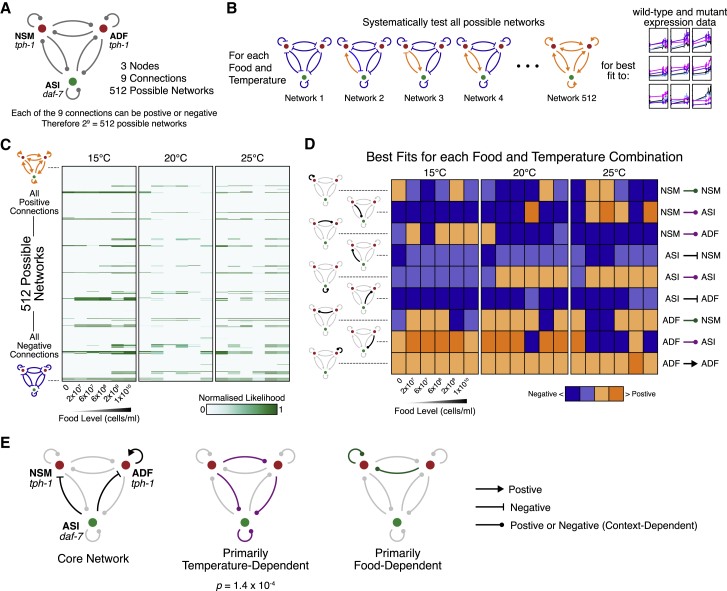
Temperature-Dependent Changes in the Multicellular *tph-1*/*daf-7* Network (A) Models of a multicellular network comprising *tph-1* in NSM and ADF and *daf-7* in ASI. (B) Identifying best-fit models of the network at each food and temperature combination, based on expression data from Figure 5. (C) Heatmap summarizing goodness of fit for all 512 models across 18 food-temperature combinations. Each row corresponds to a specific network model; each column is a food-temperature combination. (D) Summary of best-fit models for each food-temperature combination. Each edge is categorized as positive or negative regulation and as strong or weak. Each row denotes a specific connection (left and right labels); each column indicates a food-temperature combination. (E) Some regulatory connections are invariant (core network), while others change between positive and negative regulation depending on the environment. p indicates significance of the temperature-dependent configurations from bootstrapping (STAR Methods). See also related Figure S8.

To understand the temperature-dependent changes in this *tph-1*/*daf-7* network, we used *in silico* modeling to disentangle its complex interactions (Figures 6 and S8; see STAR Methods). We modeled this network as three interconnected nodes (*tph-1* in NSM, *tph-1* in ADF, and *daf-7* in ASI). Since loss of *tph-1* and *daf-7* independently affects expression of both genes in all cells, there are a total of nine effective connections that represent the net regulation of one node by another (Figure 6A). Each connection could represent positive or negative regulation, resulting in 2^9^ (=512) possible networks. Conventionally, if loss of gene A leads to increased expression of gene B, one would infer that the best fit model is gene A inhibiting gene B. We embedded this type of logic within the computational model to describe the net regulatory effects that were consistent with the observed gene expression phenotypes.

We then characterized the effects of the environment in several steps. First, considering only one food-temperature combination, we determined which of the 512 possible network models provided the best fit for the expression of both genes in all three cells across all four genotypes (Figure 6B). Next, we repeated these fits across each of the 18 food-temperature combinations (Figure 6B) and visualized how well each network configuration explains the gene expression phenotypes at each environmental combination (Figure 6C). Network configurations that fit well were sparse, indicating that our data provided sufficient constraints. Many networks that fit well were similar, as shown by the repeated spacing between the good fits within each column, which differed by the sign of only one connection. We then visualized the best-fit network for each food-temperature combination (Figure 6D) by categorizing each connection as positive or negative and as strong or weak, which revealed stable and variable connections. For example, the computational analysis suggested that *daf-7* in ASI negatively regulates *tph-1* expression in NSM and ADF across all food and temperature conditions (Figure 6D, fourth and sixth rows), consistent with experimental data showing that *daf-7(−)* mutants possessed higher *tph-1* expression in NSM and ADF than wild type in all conditions tested (Figures 5 and S5). In this manner, we used model fitting (Figures 6B and S8) as an analytical approach to identify putative regulatory interactions that were stable as well as those that primarily varied with temperature or food (Figure 6E).

### Temperature-Dependent Connections Produce Compensatory Increases in Food Responsiveness

**Figure 7:**
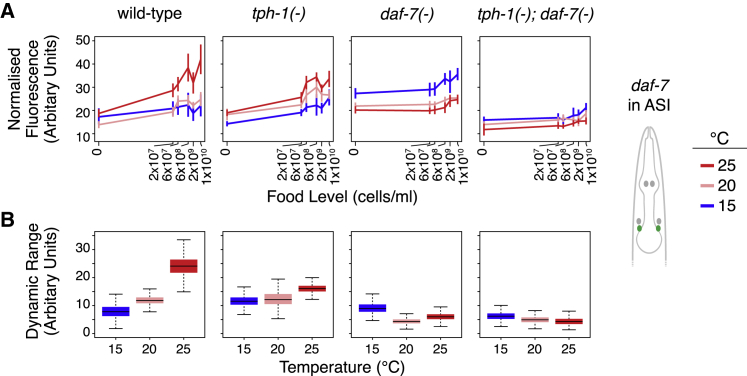
Temperature-Dependent Connections Mediate Increases in the Dynamic Range of *daf-7* with Temperature (A) Role of temperature-dependent connections converging on *daf-7* expression in ASI revealed by loss of *tph-1* or *daf-7*. Bayesian estimates for *daf-7* expression in ASI are shown; error bars indicate 90% confidence intervals (see Table S4). (B) Temperature-dependent increase in the food-responsive dynamic range of *daf-7* expression in ASI requires temperature-dependent connections that converge on *daf-7* expression in ASI (see Table S5). Bayesian distributions of dynamic range are indicated by boxplots as described in Figure 1E. Range of sample sizes for the 18 food and temperature conditions tested is as follows: wild type n = 405–2,958, *tph-1(−)* n = 70–313, *daf-7(−)* n = 90–191, *tph-1(−);daf-7(−)* n = 57–145 (please see Table S4 for detailed sample sizes).

These computational analyses pointed to the regulatory interactions that changed sign with temperature, three of which converged on *daf-7* expression in ASI (Figure 6E, center). This convergence was notable because the food-responsive dynamic range of *daf-7* expression in ASI showed the greatest temperature-dependent increase among all three cells (Figure 4D), leading us to focus on the subset of our experimental data (Figures 5 and S5 and S6) involving the regulation of *daf-7* expression in ASI (Figure 7). First, we considered the role of the cross-regulation from *tph-1* in NSM and ADF to *daf-7* in ASI by examining experimental data from the *tph-1(−)* mutant where these connections are ablated. In wild-type animals, the dynamic range of food-responsive *daf-7* expression in ASI increased with temperature (Figures 4D and 7), as highlighted by the almost non-overlapping distributions at 15°C and 25°C in Figure 7B (for quantification, see Table S5). This increase in dynamic range was impaired in *tph-1(−)* mutants (as shown by the overlapping distributions in Figure 7B), indicating that *tph-1* is required for increased food responsiveness of *daf-7* expression in ASI at higher temperatures.

Second, we considered the role of *daf-7* self-regulation by examining the experimental data from the *daf-7(−)* mutant. In wild type, expression levels of *daf-7* in ASI increased with higher temperature (Figure 7A). In *daf-7(−)* mutants, this temperature effect was reversed: *daf-7* expression in ASI decreased with higher temperature (Figure 7A). Thus, the increased expression at higher temperature was not simply due to thermodynamics; instead, it is genetically controlled. This autoregulatory effect of *daf-7* was not due to indirect feedback through *tph-1* because this inverted response to temperature was independent of *tph-1*: it was not observed in *tph-1(*−*)* mutants and still occurred when *daf-7(*−*)* was mutated in the *tph-1(−)* background (compare *tph-1(*−*)* to *tph-1(−);daf-7(−)* in Figure 7A). The *daf-7* self-regulation also contributed to increasing the dynamic range of *daf-7* expression in ASI with higher temperature, and loss of both *tph-1* and *daf-7* abolished the temperature-dependent increase in the dynamic range of food-responsive gene activity, as these values at different temperatures now overlap (Figure 7B).

These complementary results from computational analysis and experiments independently revealed roles of specific connections in the *tph-1*/*daf-7* network that vary with temperature. These temperature-dependent connections transform the way food is encoded by *daf-7* expression in ASI, from a representation of food abundance with a constant dynamic range to one where the dynamic range increases with temperature (Figure 7B). These transformations reflect specific computations performed by specific connections to compensate for the reduced impact of *tph-1* and *daf-7* on lifespan as temperature increases, thereby providing a mechanism that contributes to temperature-robust food discrimination at the level of lifespan outputs (Figures 2B and 2C).

## Discussion

Much work on robustness has focused on processes that generate a stereotyped output such as the development of specific anatomical structures or the production of precise biological rhythms (Félix and Barkoulas, 2015; Hiesinger and Hassan, 2018; Kitano, 2004; Marder et al., 2015; Mohawk et al., 2012). Here, we provide insights into temperature robustness of a responsive process where different food levels must be distinguished to produce environmentally appropriate lifespans. This type of robustness in a higher order function only became apparent when we used decoding analysis to quantify discrimination. This end-to-end robustness reflects a biologically relevant link from environment to physiology and explains the need for food and temperature responsiveness as well as for robustness to temperature. Lifespan extension at low food levels enables *C. elegans* to reproduce much later when food becomes plentiful (Angelo and Van Gilst, 2009; Templeman and Murphy, 2018). It is therefore advantageous to maintain this form of phenotypic plasticity within the reproductive temperature range, by ensuring that temperature does not interfere with the biologically critical link between food and lifespan. Although food modulates lifespan in a complex multi-step processes involving many neuroendocrine pathways that operate in diverse tissues (Alcedo et al., 2010; Bishop and Guarente, 2007; Fontana and Partridge, 2015; Greer and Brunet, 2009; Kapahi et al., 2017; Lemieux and Ashrafi, 2015; Riera and Dillin, 2016; Tao et al., 2016; Templeman and Murphy, 2018), our findings show that temperature robustness occurs at an early sensory step, because *tph-1* and *daf-7* act in sensory neurons that detect food (Cunningham et al., 2012; Entchev et al., 2015; Fletcher and Kim, 2017; Gallagher et al., 2013; Ren et al., 1996; Rhoades et al., 2019; Schackwitz et al., 1996; Sze et al., 2000; You et al., 2008; Zaslaver et al., 2015). This temperature-robust feature requires temperature responsiveness in the *tph-1*/*daf-7* network to elicit compensatory changes (Figures 6E and 7), demonstrating that plasticity to one factor is required for robustness to a second factor.

Feedback, buffering, and redundancy are robustness mechanisms that enable biological systems to compensate against perturbations (Félix and Barkoulas, 2015; Kitano, 2004). These mechanisms have been implicated in many temperature-robust processes, such as buffering by microRNAs during development (Félix and Barkoulas, 2015), feedback regulation of ion channels in neural circuit activity (O’Leary and Marder, 2016), as well as intercellular coupling at the organismal level and allosteric feedback at the molecular level in circadian oscillations (Millius and Ueda, 2017; Mohawk et al., 2012). Robustness is also tied to degeneracy, the ability of different components to impart the same function (Edelman and Gally, 2001). In degenerate systems with distinct components that are sensitive to different conditions, robustness is ensured because some components remain functional when others are perturbed (Beverly et al., 2011; Cropper et al., 2016; Marder et al., 2015). Our data suggest that *tph-1*/*daf-7* network utilizes a different implementation, where degeneracy is induced by a perturbation that reconfigures the connectivity of its components. In turn, this induced degeneracy produces similar discriminatory performance under different conditions to generate robustness to the perturbation.

Three principal implications arise from our finding that different environments induce different network configurations to produce the same performance. First, biological networks in general, and gene regulation in particular, are not static. It will be particularly intriguing to understand the context-dependent organization of many biological networks to learn how their changes relate to their functions in complex environments. Second, robustness can be genetically controlled. We reveal that serotonin and TGF-β signals impinge on the robustness of food sensing to temperature. These results align with precedents uncovering important roles for neuromodulators in temperature-robust rhythmic neural activity and thermosensory behavior (Beverly et al., 2011; Haddad and Marder, 2018). Third, gene-environment interactions are a by-product of induced degeneracy, where under different conditions, network components possess different functions and therefore exhibit different loss-of-function phenotypes. If induced degeneracy is a common robustness mechanism, we might expect studies of gene-environment interactions to highlight robustness in additional areas of biology.

## STAR★Methods

### Key Resources Table

**Table:** 

REAGENT or RESOURCE	SOURCE	IDENTIFIER
**Bacterial and Virus Strains**		

*E. coli:* OP50	*Caenorhabditis* Genetics Center	WB Strain: OP50

**Chemicals, Peptides, and Recombinant Proteins**		

Bacto Agar	BD Biosciences	214030
Bacto Peptone	BD Biosciences	214931
LB Broth Miller	BD Biosciences	241410
Sodium Chloride	Sigma-Aldrich	S9888
Magnesium Sulfate	Sigma-Aldrich	M7506
Calcium Cholride	Sigma-Aldrich	C1016
Potassium Phosphate monobasic	Sigma-Aldrich	P5655
Potassium Phosphate dibasic	Sigma-Aldrich	P2222
Isopropyl-b-D-thiogalactopyranoside (IPTG)	Sigma-Aldrich	I5502
Carbenicillin disodium	Sigma-Aldrich	C9231
Kanamycin sulfate	Sigma-Aldrich	60615

**Deposited Data**		

*C. elegans* lifespan data	This Study	Tables S1, S2, and S3
*C. elegans* gene expression data	This Study	Tables S4, S5, and S6

**Experimental Models: Organisms/Strains**		

*C. elegans*: N2 Bristol	*Caenorhabditis* Genetics Center	WB Strain: N2
*C. elegans*: QL101 *tph-1(n4622) II*	This study; Entchev et al., 2015	N/A
*C. elegans*: QL282 *daf-7(ok3125) III*	This study; Entchev et al., 2015	N/A
*C. elegans*: QL300 *tph-1(n4622) II; daf-7(ok3125) III*.	This study; Entchev et al., 2015	N/A
*C. elegans*: QL196 *drcSi61[tph-1p::mCherry, Cbr-unc-119(+)] I; drcSi7[daf-7p::Venus, Cbr-unc-119(+)] II*	This study; Entchev et al., 2015	N/A
*C. elegans*: QL404 *drcSi61[tph-1p::mCherry, Cbr-unc-119(+)] I; tph-1(n4622) drcSi7[daf-7p::Venus, Cbr-unc-119(+)] II*	This study; Entchev et al., 2015	N/A
*C. elegans*: QL402 *drcSi61[tph-1p::mCherry, Cbr-unc-119(+)] I; drcSi7[daf-7p::Venus, Cbr-unc-119(+)] II; daf-7(ok3125) III*	This study; Entchev et al., 2015	N/A
*C. elegans*: QL435 *drcSi61[tph-1p::mCherry, Cbr-unc-119(+)] I; tph-1(n4622) drcSi7[daf-7p::Venus, Cbr-unc-119(+)] II; daf-7(ok3125) III*	This study; Entchev et al., 2015	N/A

**Recombinant DNA**		

Ahringer *C. elegans* RNAi library: RNAi *egg-5* knockdown: pL4440-egg-5	Source Bioscience	https://www.sourcebioscience.com/products/life-sciences-research/clones/rnai-resources/c-elegans-rnai-collection-ahringer/

**Software and Algorithms**		

LabView	National Instruments	https://www.ni.com/en-us/shop/labview.html
MATLAB	Mathworks	https://www.mathworks.com/products/matlab.html
R	R Core Team	https://www.r-project.org
Rjags package for R	Martyn Plummer, Alexey Stukalov and Matt Denwood	http://mcmc-jags.sourceforge.net

### Resource Availability

#### Lead Contact

Further information and requests for resources and reagents should be directed to and will be fulfilled by the Lead Contact, QueeLim Ch’ng (queelim@kcl.ac.uk)

#### Materials Availability

All strains reported in this study are available on request from the lead contact.

#### Data and Code Availability

All lifespan data are available in full on request from the lead contact. The code for quantification of the fluorescence signals from ASI, ADF, and NSM neurons is publicly available on GitHub (https://github.com/meizhan/SVMelegans). Custom R scripts and data used in the Bayesian analyses of lifespan (Tables S1, S2, and S3) and gene expression (Tables S4, S5, and S6) are publicly available on GitHub (https://github.com/giovannidiana/TRFS). The custom R scripts used in the decoding analyses are publicly available on GitHub (https://github.com/giovannidiana/TRFS).

### Experimental Model and Subject Details

#### *C. elegans* Strains and General Culture

All strains of *C. elegans* were cultured using standard conditions (Stiernagle, 2006). The following strains were used for the lifespan analyses: N2 (wild-type), QL101 *tph-1(n4622) II*, QL282 *daf-7(ok3125) III*, and QL300 *tph-1(n4622) II; daf-7(ok3125) III*. For quantitative imaging analyses the following strains were used: QL196 *drcSi61[tph-1p::mCherry, Cbr-unc-119(+)] I; drcSi7[daf-7p::Venus, Cbr-unc-119(+)] II*, QL404 *drcSi61[tph-1p::mCherry, Cbr-unc-119(+)] I; tph-1(n4622) drcSi7[daf-7p::Venus, Cbr-unc-119(+)] II*, QL402 *drcSi61[tph-1p::mCherry, Cbr-unc-119(+)] I; drcSi7[daf-7p::Venus, Cbr-unc-119(+)] II; daf-7(ok3125) III*, and QL435 *drcSi61[tph-1p::mCherry, Cbr-unc-119(+)] I; tph-1(n4622) drcSi7[daf-7p::Venus, Cbr-unc-119(+)] II; daf-7(ok3125) III*. All experiments were conducted using hermaphrodite animals and the age or developmental stage of animals is detailed in the section below.

### Method Details

#### Lifespan Studies

Lifespans were carried out using hermaphrodite worms as previously described (Entchev et al., 2015; Patel et al., 2017) (Figure 1A). Briefly, starting with starved animals, animals were raised for two generations on standard NGM culture plates (Stiernagle, 2006) seeded with the live OP50 at the baseline temperature of 20°C. The L4-stage progeny of the F2 generation were then transferred to RNAi plates seeded with bacteria expressing double-stranded RNA (dsRNA) targeting the *egg-5* gene, which is critical for egg-shell formation (Cheng et al., 2009; Parry et al., 2009), for 24 h at 20°C. This short period of exposure to *egg-5* RNAi is sufficient to prevent progeny production in the vast majority of animals throughout their reproductive period and does not affect how food modulates lifespan (Entchev et al., 2015; Patel et al., 2017). This step is especially necessary for *tph-1(-)* and *daf-7(-)* mutants, as well as the double mutant of these genes, as they have an impaired egg-laying phenotype which results in the premature death of the adult worm from matricide. After the 24-hour *egg-5* RNAi exposure, the 1-day old adults were moved to NGM culture plates supplemented with the antibiotics streptomycin and carbenicillin (NSC) seeded with our baseline food level, antibiotic-inactivated OP50 at a concentration of 2x10^9^ cells/ml (Entchev et al., 2015; Patel et al., 2017), for 24 h at 20°C. All manipulations away from the baseline temperature and food level occurred on day 2 of adulthood (Figure 1A). In total, we examined lifespan across 4 different temperatures and 6 different food levels (Figure 1B). Animals at each temperature were manually transferred to new NSC plates seeded with the appropriate food concentration according to the schedule laid out previously (Patel et al., 2017). Animals were scored for death at every transfer point and then daily after the final transfer point for each temperature. Death was assessed as the failure to detect movement in response to a gentle prod with a wire pick. Part of the raw data for lifespans at 20°C was obtained during our previous study (Entchev et al., 2015).

#### Quantitative Fluorescence Imaging

Gene expression activity of *daf-7* and *tph-1* at single-cell and single-worm resolution were quantified based on the fluorescence intensity of *daf-7::Venus* and *tph-1::mCherry* transcriptional reporters as previously described (Entchev et al., 2015). These reporters were integrated in single-copy and contain endogenous 5′ and 3′ sequences up to the next adjacent open reading frame; and their construction and validation were detailed previously (Entchev et al., 2015). Multiple labs have validated that transcriptional reporter fluorescence correlates with downstream activity in these respective pathways (Entchev et al., 2015; Fletcher and Kim, 2017; Ren et al., 1996; Zhang et al., 2005).

For quantitative imaging, strains were cultured using a procedure similar to that in lifespan studies (Entchev et al., 2015; Patel et al., 2017). Animals were initially raised on large NGM plates (10 cm diameter) for two generations. Once the F2 animals reached reproductive adulthood, they were washed from the plates using S-basal supplemented with streptomycin and then subjected to a sodium hypochlorite treatment to break them apart and release their eggs (Stiernagle, 2006). The eggs were then deposited on to new large NGM plates seeded with live OP50 and left to hatch and grow at 20°C till the animals reached the L4-stage. The *daf-7(ok3125)* mutation causes animals to constitutively enter a transient dauer arrest (Entchev et al., 2015; Patel et al., 2017). For this reason, strains harboring *daf-7(ok3125)* were prepared 24 h before strains without this mutation, to ensure sufficient numbers of animals exited dauer and reached the L4-stage. Once animals reached the L4-stage, they were collected by washing and distributed on to large RNAi plates seeded with *egg-5*-dsRNA expressing bacteria at a density of ∼100 worms per plate. Animals were exposed to *egg-5* RNAi for 1 day at 20°C before being moved to large NSC plates seeded with our baseline food level for a further 24 h also at 20°C. As with the lifespan studies, all temperature and food manipulations were initiated on day 2 of adulthood. Worms at each temperature were transferred by washing to new large NSC plates seeded with the appropriate food concentration according to the schedule laid out previously (Patel et al., 2017). Animals were harvested for imaging upon reaching their sixth day of adulthood.

Imaging studies were performed on the microfluidic system previously described (Entchev et al., 2015). Briefly, our imaging system consists of a custom two-layer microfluidic device (Chung et al., 2008; Crane et al., 2012) constructed from polydimethylsiloxane (PDMS) using standard multilayer soft lithographic techniques (Unger et al., 2000) and bonded to a glass coverslip. Animals were loaded into the imaging area of the device using pressure driven flow controlled by on-chip valves that reside in the first layer of the device. The second layer of the device cooled the imaging area down to ∼4°C, which immobilizes the worms during image capture. Images of the head of animal were captured as Z stacks in 2 μm steps using a Nikon Ti-Eclipse inverted scope with a 40x (1.3 NA) oil objective on a Hamamatsu Orca R2 camera. The fluorescence intensities of the Venus and mCherry reporters was captured simultaneously using an Optosplit II system. Operation of worm-loading on the microfluidic system and subsequent image acquisition was automated through the use of a custom LabView code. Images were stored locally for offline processing using a custom MATLAB script that automatically assigned cell ID and extracted fluorescent intensities for the ASI, ADF, and NSM neurons in each stack. Part of the raw data for gene expression at 20°C was obtained during our previous study (Entchev et al., 2015).

### Quantification and Statistical Analysis

#### General Statistical Analysis

To account for biological and experimental variability between and within experiments, we adopted a Bayesian framework to analyze the lifespan and gene expression data. We used hierarchical models to describe these sources of variability, and Bayesian inference to estimate the full probability distributions (so-called posterior distributions) of features such as mean lifespan and expression levels given the trial-to-trial and within trial variability. Throughout these analyses we used non-informative (flat) priors for all model parameters to minimize their effect on posterior distributions.

To compare two measurements (e.g., mean lifespan at two different conditions), one can directly examine the amount of overlap between their posterior distributions, which reflects the statistical confidence of their difference. Non-overlapping posterior distributions imply that those two measurements are statistically different. Such Bayesian comparisons are more nuanced and informative than conventional statistical tests (Kruschke, 2013), allowing us to directly estimate differences among conditions without the need of conventional tests which would require the introduction of null hypothesis and their inherent assumptions (typically Gaussian) on the noise when calculating p values. This approach also has the advantage of not being subject to misconceptions and pitfalls associated with interpretation of p values (Wasserstein and Lazar, 2016).

The range of the posterior distributions are visualized throughout the figures with boxplots plotted with the standard “boxplot” function in R. These plots directly show which distributions do not overlap and are therefore statistically distinct, such as the dynamic range of lifespan across different food levels at 15°C versus 20°C in the right part of Figure 1E; and the food decoding power in wild-type versus *tph-1(-); daf-7(-)* double mutants at all temperatures in Figure 3B. Additionally, we employed the posterior samples of model parameters to estimate the overlaps in various measurements across food/temperature conditions (Tables S1, S2, S3, S4, S5, and S6). We calculated each variable of interest (dynamic range, mean values, etc.) corresponding to each combination of parameters representing the posterior distributions to ensure consistent error propagation (below). Therefore, to compare the distribution of a given variable between conditions A and B, we quantified the fraction *p* of the random samples where that variable was larger in A. The value in our comparison table is obtained as the minimum between *p* and 1-*p* and range from 0 (no overlap between distributions) to 0.5 (100% overlap between distributions).

#### Bayesian Analysis of Lifespan

We modeled lifespan as a Weibull distribution with trial-dependent scale and shape parameters to accommodate batch variability. To estimate the average lifespan for each environmental condition with censoring using a data augmentation technique, we used the RJAGS package in R (Plummer, 2018).

#### Bayesian Analysis of Gene Expression

To estimate *daf-7* and *tph-1* expression levels in their respective cells across all our experiments, we designed a Bayesian approach to incorporate all sources of experimental and biological variability within a unified hierarchical model (Figure S8A). This model allows us to simultaneously quantify the effect of trial-to-trial variability, population variability, changes in LED illumination, and levels of correlation among *tph-1* and *daf-7* in ADF, ASI, and NSM. To correct for changes in LED illumination, we periodically imaged fluorescent bead standards in both red and green channels and used linear regression of these measured values over time to obtain normalization factors for each experiment. The posterior distributions of these regression coefficients were calculated as the product of an inverse gamma distribution associated with the time-independent variance in bead fluorescence and the multivariate normal distribution of their fluorescence (Carlin and Louis, 2009). For each food and temperature condition, the unnormalized mean batch fluorescence $F_{b,n}^{\left(1\right)}$ for cell *n* of batch *b* imaged at time $\tau_{b}$ is expressed as:

$$F_{b,n}^{\left(1\right)}=\;f_{b,n}^{\left(1\right)}\cdot \left(C_{1}\;+\;C_{2}\cdot \;\tau_{b}\right)$$

where *C*_*1*_ and *C*_2_ are the linear regression coefficients for the relevant imaging channel modeled statistically according to the posterior distributions conditional to bead measurements, and $f_{b,n}^{\left(1\right)}$ is the batch-level normalized fluorescence for each neuron. Normalized fluorescence levels for all cells are modeled with multivariate normal distributions as described in Figure S8A. We implemented this model using the RJAGS package in R (Plummer, 2018) which performs Monte Carlo Gibbs sampling and provides the posterior distribution of all model variables given the raw data, allowing us to estimate the true fluorescence values and its uncertainty. The Gibbs sampler returns a list of values (Markov chains) for each model variable distributed according to their corresponding posterior distributions. The convergence of the sampler was estimated by requiring the convergence of 8 independent Markov chains to the same set of model parameters.

#### Decoding Analysis

To measure discrimination between different food and temperature conditions based on either expression or lifespan (Figure S8B), we first expressed the probability of each response under each environmental condition based on the Bayesian analysis above. The maximum-likelihood decoder attributes each response to the environmental stimuli that maximizes the conditional probability. By applying the maximum-likelihood decoder to the data from all individuals, we can calculate the confusion matrix (Figures 2, 3, S2, and S7) by counting how many times a response measured under a given environment is decoded as the correct environment, or as one of the incorrect environments. The closer the confusion matrix to a diagonal matrix, the more informative the response is about the state of the environment (Figure S8B). To apply the maximum-likelihood decoder to our gene expression models, we estimated the probability distribution of the normalized expression levels for all environmental conditions. Because the normalization uncertainty is small compared to population and trial-to-trial variability, we approximated these distributions as multivariate normal distributions with means equal to the global mean fluorescence (top level of hierarchical model, Figure S8A) and with a covariance matrix obtained by adding quadratically the variances from all layers of the hierarchical model. We previously showed that maximum-likelihood decoding and other analysis methods yield similar conclusions, indicating that our results are not sensitive to the analytical technique used (Diana et al., 2017; Entchev et al., 2015).

#### Network Model of tph-1 and daf-7 Regulation

In this section we discuss our modeling approach for characterizing the regulatory interactions among neurons. As a short-hand notation we will denote the *ADF*, *ASI* and *NSM* neurons as *x*, *y,* and *z* respectively. Here we introduce a computational method to characterize neuronal regulatory interactions based on the ratios between expression levels in mutants and wild-type. We assigned three binary variables: $s_{x}$, $s_{y}$ and $s_{z}$ to all neurons and then considered the 8 configurations generated by the triplet $(s_{x}$, $s_{y},s_{z})$ as in Figure S8C (right panel).

We embedded these 8 configurations into the cubic network depicted in Figure S8C (left panel) allowing transitions between configurations that only differ by one of the binary variables. We then introduce parameters representing the level of interaction among neurons to define the transitions between configurations. The probabilities for each configuration can be interpreted as a representation of the circuit under a given choice of interaction parameters.

Given a transition rate matrix *W*, the probability vector of the 8 configurations $P=\left\{p_{1},\cdots ,p_{8}\right\}$ satisfies the Master equation (van Kampen, 2007)

(S1) $$\frac{dp_{i}}{d\tau }=\;\underset{j\neq i}{\sum }\left(W_{ij}p_{j}-W_{ji}p_{i}\right)\;$$

where the first term on the right hand side is a positive contribution due to incoming transitions from any node *j* connected to node *i* and a negative contribution due to outgoing transitions from node *i*. Note that the matrix element $W_{ij}$ denotes the rate of the transition *j* → *i*. The steady-state probabilities are then obtained by solving the linear equation $\underset{j\neq i}{\sum }\left(W_{ij}p_{j}-W_{ji}p_{i}\right)=\;0$.

To parameterize the transition rates, we introduced 9 parameters representing 3 self-regulations and 6 cross-regulations summarized by the regulatory matrix *w*

(S2) $$w\;=\;\left(\begin{array}{lll} w_{xx} & w_{xy} & w_{xz}\\ w_{yx} & w_{yy} & w_{yz}\\ w_{zx} & w_{zy} & w_{zz} \end{array}\right)=\;\left(\begin{array}{lll} t_{1} & t_{2} & t_{3}\\ t_{4} & t_{5} & t_{6}\\ t_{7} & t_{8} & t_{9} \end{array}\right)$$

where each matrix element $w_{\alpha \beta }$ characterizes the negative regulation of neuron β due to neuron α. To keep the analysis general, we assumed non-symmetric mutual regulation between neurons, i.e., $w_{\alpha \beta }\neq \;w_{\beta \alpha }$. By using this regulatory matrix, we can parameterize the transition rate matrix *W* as

(S3) $$W=\;\left(\begin{array}{llllllll} 0 & w_{xx} & w_{yy} & 0 & w_{zz} & 0 & 0 & 0\\ k_{on} & 0 & 0 & w_{xy}w_{yy} & 0 & w_{xz}w_{zz} & 0 & 0\\ k_{on} & 0 & 0 & 0 & 0 & w_{yz}w_{zz} & w_{yz}w_{zz} & 0\\ 0 & k_{on} & k_{on} & 0 & 0 & 0 & 0 & w_{xz}w_{yz}w_{zz}\\ k_{on} & 0 & 0 & 0 & 0 & w_{zx}w_{xx} & w_{zy}w_{yy} & 0\\ 0 & k_{on} & 0 & 0 & k_{on} & 0 & 0 & w_{yy}w_{xy}w_{zy}\\ 0 & 0 & k_{on} & 0 & k_{on} & 0 & 0 & w_{xx}w_{yx}w_{zx}\\ 0 & 0 & 0 & k_{on} & 0 & k_{on} & k_{on} & 0 \end{array}\right)$$

where $k_{on}$ is the rate at which each neuron switches to the ON state. For instance, let us consider the transition from configuration 2 to configuration 1 in Figure S8C where ADF goes from an active to inactive state. The corresponding transition rate $W_{12}\;=\;w_{xx}$ reflects the role of $w_{xx}$ in parameterizing the negative (self-)regulation of ADF. When $w_{xx}$ is large, the transitions 2 *→* 1 are enhanced by the regulation, which reduces the (steady state) probability of ADF being active. Vice-versa, when $w_{xx}$ is small, configuration 2 is more stable. Now, let us consider as a second example, the transition from configuration 4 =(1,1,0) where ADF and ASI are both ON, to configuration 2 =(1,0,0) where ASI has been switched OFF. The corresponding transition rate is $W_{24}\;=\;w_{xy}w_{yy}$. In this case the rate is affected by both ASI itself through the self-regulation term $w_{yy}$ and ADF through $w_{\text{xy}}$.

By setting the reference rate $k_{on}=\;1$, all terms in the regulatory matrix that are larger than 1 generate downregulation (in the case of cross-regulatory terms) or are smaller than $k_{on}$ (in the case of self-regulatory terms).

Given a set of regulatory parameters *w*’s, we can obtain the probability of each configuration by considering the steady state of the Master equation dynamics. We can also derive the (marginal) probabilities of each neuron being in an active state from all configurations where that neuron is active:

(S4) $$Q_{x}\;=p_{2}\;+\;p_{4}\;+\;p_{6}\;+\;p_{8}$$

(S5) $$Q_{y}\;=p_{3}\;+\;p_{4}\;+\;p_{7}\;+\;p_{8}$$

(S6) $$Q_{z}\;=p_{5}\;+\;p_{6}\;+\;p_{7}\;+\;p_{8}$$

With this setting, we can mimic the effect of null mutant of a gene by removing from the network all edges arising from that gene. For instance, we can model the *daf-7(-)* mutant by removing edges $t_{4}$, $t_{5}$ and $t_{6}$ as shown in Figure S8D. Note that edges $t_{2}$ and $t_{8}$ are still in the network because in the *daf-7(-)* mutant the promoter is still present. The removal of an edge from the network is equivalent to setting the corresponding regulatory interaction w equal to 1. At fixed regulatory parameters, we can then compare how the probabilities of *ADF*, *ASI* and *NSM* being active change between wild-type and mutant networks. We can define the ratios between the probability of each neuron being active in wild-type and each mutant condition as

(S7) $${\bar{R}}_{n}^{\left(1\right)}\equiv \frac{Q_{n}^{daf-7\left(-\right)}}{Q_{n}^{wt}}$$

(S8) $${\bar{R}}_{n}^{\left(2\right)}\equiv \frac{Q_{n}^{tph-1\left(-\right)}}{Q_{n}^{wt}}$$

(S9) $${\bar{R}}_{n}^{\left(3\right)}\equiv \frac{Q_{n}^{tph-1\left(-\right);\;daf-7\left(-\right)}}{Q_{n}^{wt}}$$

where n = *x, y, z*. To fit the regulatory interactions, we have constrained these quantities by using the same ratios obtained from the expression levels

(S10) $$R_{x}^{\left(1\right)}=\;\frac{F_{x}^{daf-7\left(-\right)}}{F_{x}^{wt}},\;R_{y}^{\left(1\right)}=\;\frac{F_{y}^{daf-7\left(-\right)}}{F_{y}^{wt}},\;R_{z}^{\left(1\right)}=\;\frac{F_{z}^{daf-7\left(-\right)}}{F_{z}^{wt}}$$

(S11) $$R_{x}^{\left(2\right)}=\;\frac{F_{x}^{tph-1\left(-\right)}}{F_{x}^{wt}},\;R_{y}^{\left(2\right)}=\;\frac{F_{y}^{tph-1\left(-\right)}}{F_{y}^{wt}},\;R_{z}^{\left(2\right)}=\;\frac{F_{z}^{tph-1\left(-\right)}}{F_{z}^{wt}}$$

(S12) $$R_{x}^{\left(3\right)}=\;\frac{F_{x}^{tph-1\left(-\right);\;daf-7\left(-\right)}}{F_{x}^{wt}},\;R_{\text{y}}^{\left(3\right)}=\;\frac{F_{y}^{tph-1\left(-\right);\;daf-7\left(-\right)}}{F_{y}^{wt}},\;R_{z}^{\left(3\right)}=\;\frac{F_{z}^{tph-1\left(-\right);\;daf-7\left(-\right)}}{F_{z}^{wt}}$$

To estimate these ratios from the raw data, we paired imaging experiments performed on mutants with their corresponding wild-type control, which were always performed on the same day. For all matching pairs, we estimated the mean expression levels for each neuron. We then obtained the average $<R_{n}^{\left(f\right)}>$ and the standard deviation $\sigma_{n}^{\left(f\right)}$of the three mutant/wild-type ratios across all paired experiments.

To explore the parameter space, we employed a stochastic minimization of the cost function

(S13) $$H\left(w\right)\;=\underset{n\in \left\{x,y,z\right\}}{\sum }\overset{3}{\underset{f=1}{\sum }}{\left[\frac{{\bar{R}}_{n}^{\left(f\right)}-R_{n}^{\left(f\right)}}{\sigma_{n}^{\left(f\right)}}\right]}^{2}$$

which depends on the set of regulatory parameters *w*. The algorithm proposes sequentially new parameters in the neighborhood of the previous ones (according to Euclidian distance) and accepts them if their corresponding cost function is reduced.

By performing this analysis, we found the sets of parameters that best represent the regulation among all three cells for each environmental condition of food and temperature tested (Figure 6D). This result allowed us to identify regulations that are affected by temperature (Figures 6D–6E). We showed that these conclusions are robust in two ways. First, we used alternative methods of determining the network configurations. The same temperature-dependent regulations were identified when using the geometric means of the regulatory parameters weighted by goodness-of-fit, or when using the geometric means of the regulatory parameters for all models with a normalized likelihood greater than 95%. Second, the temperature-dependence did not arise by chance alone. In 100,0000 shuffles of the environmental conditions (i.e., shuffling the columns in Figure 6D), monotonic temperature-dependent changes of the same magnitude for 4 edges occurred only in 14 permutations (Figure 6E). Together, these computational analyses independently point to the key regulations that provide robustness to temperature.

## Supplemental Information

Document S1. Figures S1–S8

Table S1. Bayesian Analyses of Lifespan across All Genotypes and Conditions, Related to Figures 1 and S2

Table S2. Quantification of the Overlap between the Dynamic Ranges of All Lifespans, Related to Figure 1

Table S3. Quantification of the Overlap between the Food Decoding Power of All Lifespans, Related to Figures 2 and 3

Table S4. Bayesian Analyses of Transcriptional Reporter Expression across All Genotypes and Conditions, Related to Figures 4, 5, and S3–S5

Table S5. Quantification of the Overlap between the Dynamic Ranges of Reporter Expression across All Conditions, Related to Figures 4, 7, and S6

Table S6. Quantification of the Overlap between the Food Decoding Power of Combinatorial Reporter Expression across All Conditions, Related to Figures 4 and S6

Document S2. Article plus Supplemental Information
