# Supplementary material for: A Multicellular Network Mechanism for Temperature-Robust Food Sensing
Source: Cell Rep. 2020 Dec 22;33(12):108521. doi: 10.1016/j.celrep.2020.108521 (PMC7773553; doi:10.1016/j.celrep.2020.108521)
Supplement: Document S1. Figures S1–S8 [file mmc1.pdf]

Cell Reports, Volume *33*

Supplemental Information

A Multicellular Network Mechanism

for Temperature-Robust Food Sensing

Dhaval S. Patel, Giovanni Diana, Eugeni V. Entchev, Mei Zhan, Hang Lu, and QueeLim Ch'ng

Figure S1

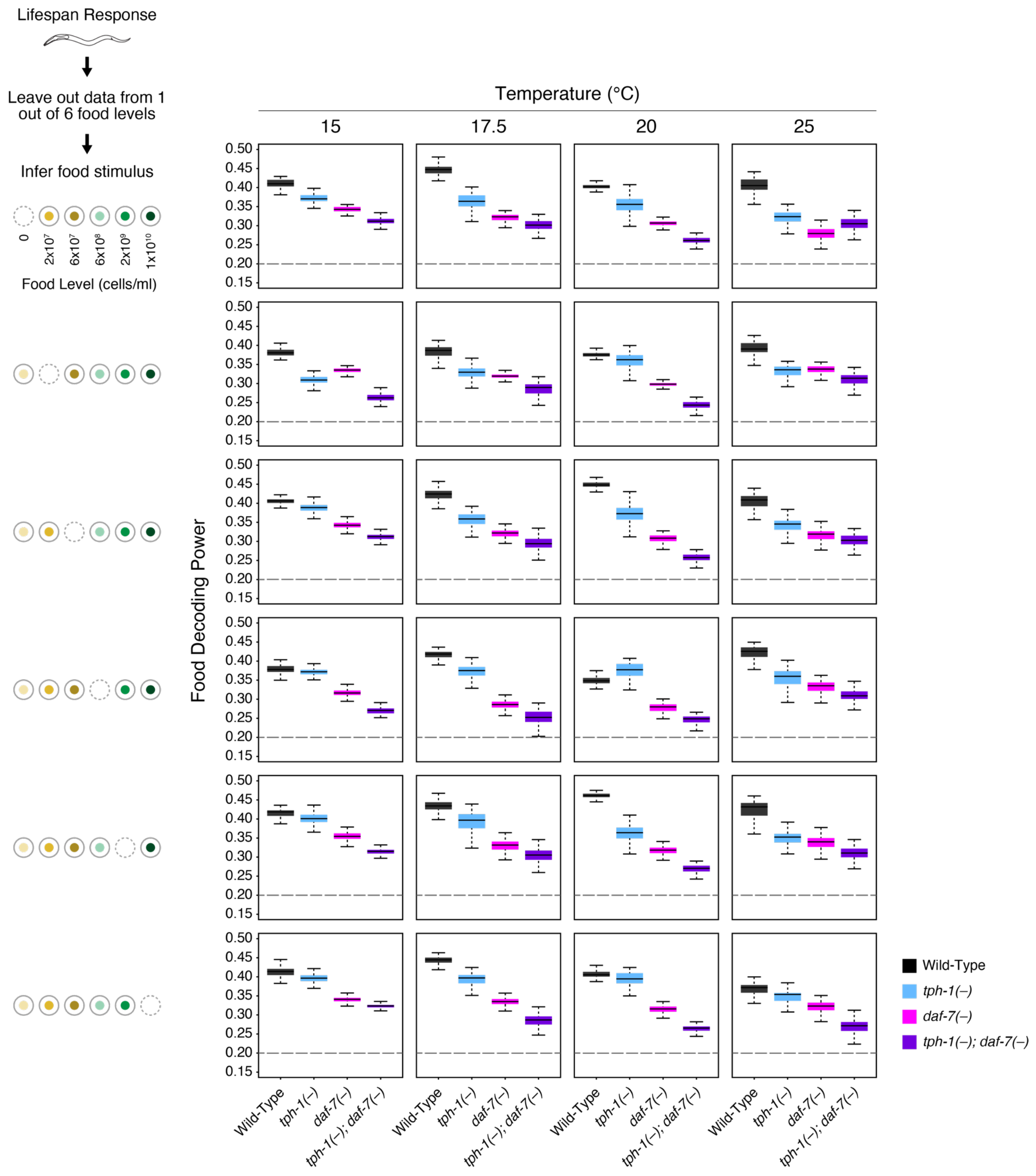

Lifespan Response
Leave out data from 1 out of 6 food levels
Infer food stimulus
0
2x10^7
6x10^7
6x10^8
2x10^9
1x10^10
Food Level (cells/ml)
Temperature (°C)
15
17.5
20
25
Food Decoding Power
0.50
0.45
0.40
0.35
0.30
0.25
0.20
0.15
Wild-Type
tph-1(–)
daf-7(–)
tph-1(–); daf-7(–)

Figure S1. Effect of temperature on food-responsiveness is not sensitive to the food levels tested. Related to Figure 3. The analysis from Figure 3B is repeated in a "leave one out" approach. In each row, one of the 6 food levels in the original dataset is left out (indicated by the dotted outline on the left) and the food decoding power computed from the remaining 5 food levels for each genotype at each temperature. Range of sample sizes for the 24 food and temperature conditions tested are as follows: wild-type n = 112 to 525, *tph-1(-)* n = 84 to 209, *daf-7(-)* n = 142 to 214, *tph-1(-); daf-7(-)* n = 130 to 213 (see Table S1 for detailed sample sizes). Bayesian distributions are depicted by boxplots as described for Figure 1E. Dotted lines indicate decoding power from random chance alone.

Figure S2

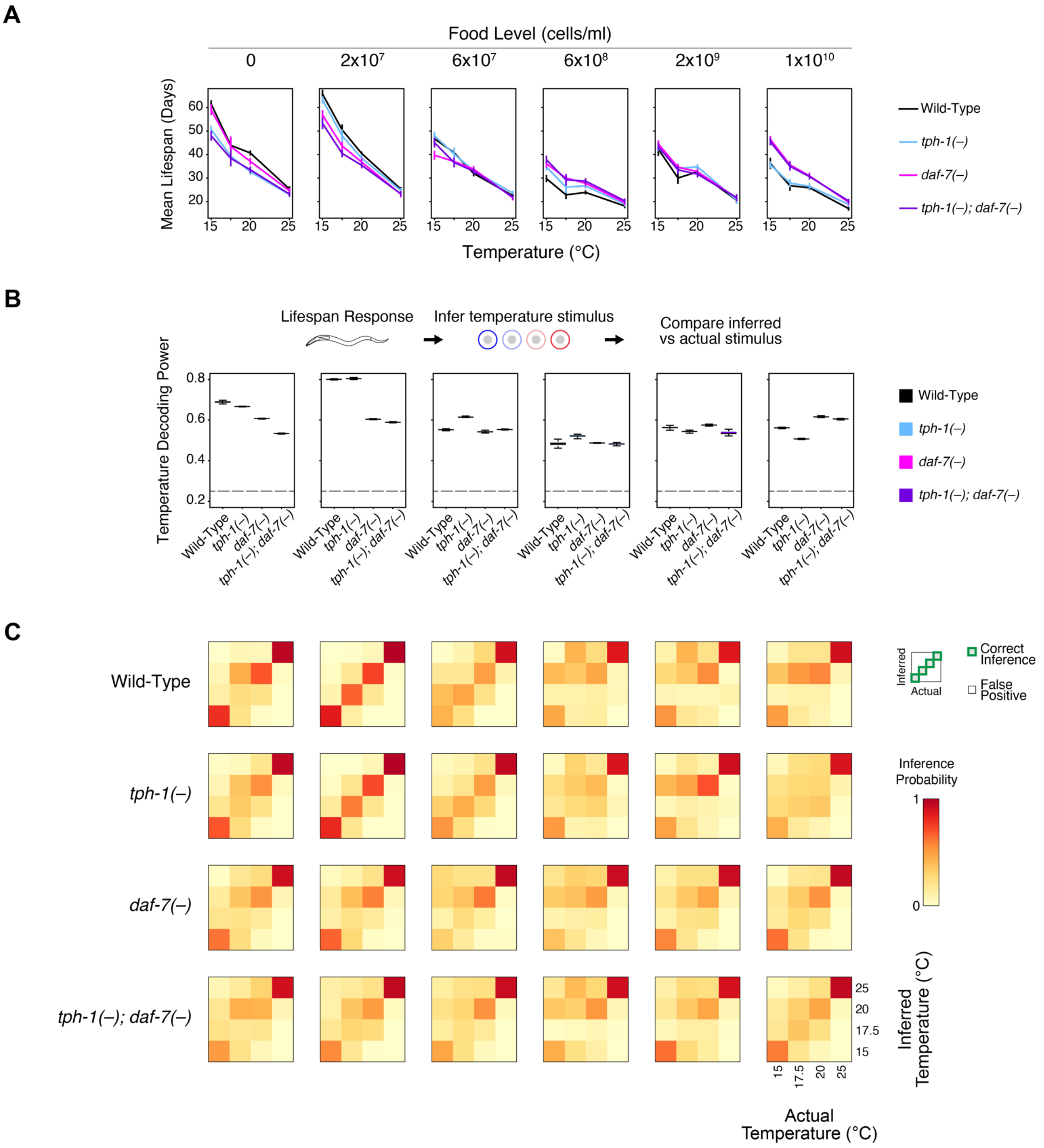
A
Food Level (cells/ml)
0
2x10^7
6x10^7
6x10^8
2x10^9
1x10^10
Mean Lifespan (Days)
Temperature (°C)
Wild-Type
tph-1(–)
daf-7(–)
tph-1(–); daf-7(–)
B
Lifespan Response
Infer temperature stimulus
Compare inferred vs actual stimulus
Temperature Decoding Power
Wild-Type
tph-1(–)
daf-7(–)
tph-1(–); daf-7(–)
C
Wild-Type
tph-1(–)
daf-7(–)
tph-1(–); daf-7(–)
Inferred
Actual
Correct Inference
False Positive
Inference Probability
Inferred Temperature (°C)
Actual Temperature (°C)

Figure S2. Effect of food on temperature-responsiveness in lifespan. Related to Figure 3. (A) Mean lifespan for each genotype as a function of temperature at each food level. Bayesian estimates are shown for all mean lifespans (see Star Methods and Table S1). Error bars indicate standard deviations. **(B)** Top: summary of temperature decoding. Bottom: Effect of *tph-1(-)* and *daf-7(-)* on temperature discrimination based on lifespan. Bayesian distributions are depicted by boxplots as described for Figure 1E. Dotted lines indicate decoding power from random chance alone. **(C)** A grid of confusion matrices depicting temperature inference at different food levels in different genotypes. Range of sample sizes for the 24 food and temperature conditions tested are as follows: wild-type n = 112 to 525, *tph-1(-)* n = 84 to 209, *daf-7(-)* n = 142 to 214, *tph-1(-); daf-7(-)* n = 130 to 213 (see Table S1 for detailed sample sizes).

Figure S3

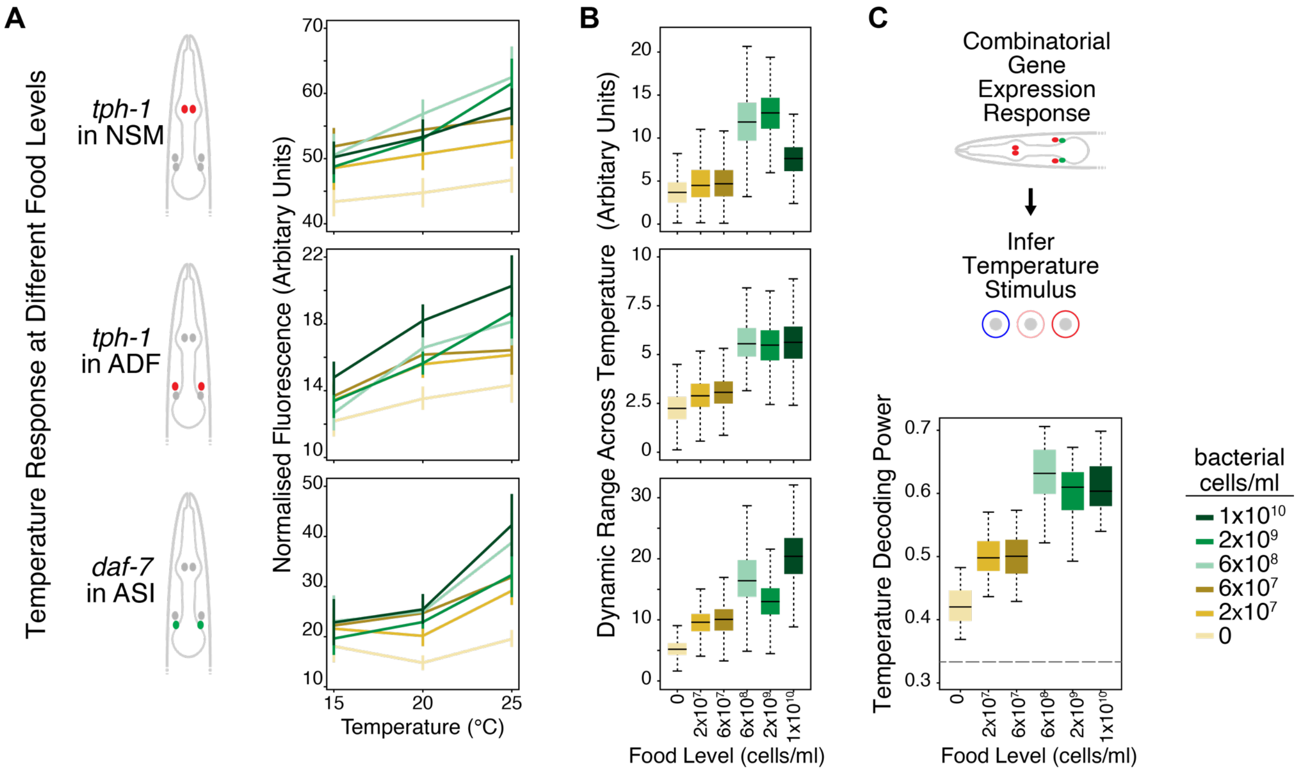

Figure S3. Effect of food on temperature responsiveness of *tph-1* and *daf-7* expression. Related to Figure 4. (A) Wild-type expression of *tph-1* in NSM and ADF, and *daf-7* in ASI as a function of temperature. Each line corresponds to a different food level. Diagrams on the left indicate the gene and cell. Legend at the right indicate food level. **(B)** Temperature-responsive dynamic range in the expression of *tph-1* in NSM and ADF, and *daf-7* in ASI at different food levels. **(C)** Wild-type temperature decoding power at different food levels based on the combinatorial expression of *tph-1* and *daf-7* in these three cells, with the dotted line indicating decoding power from random chance alone. For wild-type, n = 405 to 2958 for the 18 food and temperature conditions tested; see Table S4 for detailed sample sizes. Bayesian estimates are shown for all mean expression values and dynamic ranges (see Star Methods and Tables S4-S6). Bayesian distributions in (B)-(C) are depicted by boxplots as described for Figure 1E.

Figure S4

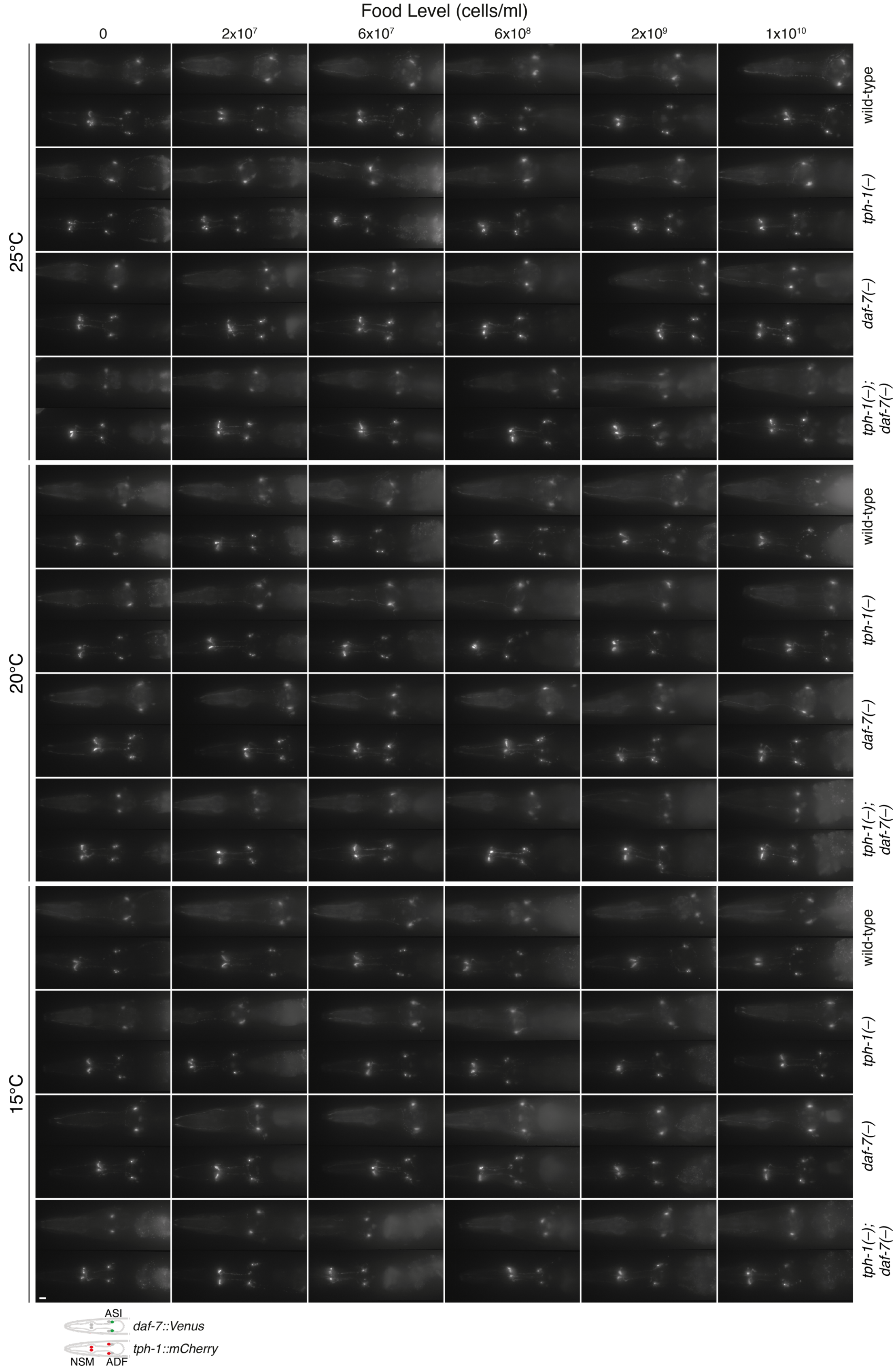

Food Level (cells/ml)
0
2x10^7
6x10^7
6x10^8
2x10^9
1x10^10
25°C
20°C
15°C
wild-type
tph-1(–)
daf-7(–)
tph-1(–); daf-7(–)
ASI
daf-7::Venus
tph-1::mCherry
NSM
ADF

Figure S4. Effect of temperature on *tph-1* and *daf-7* expression as a function of food level. Related to Figure 5. Representative images of *daf-7::Venus* (top) and *tph-1::mCherry* expression at each food level, temperature, and for each genotype. Each image is the animal that was among the closest to the population mean for expression in ASI (*daf-7::Venus*), ADF and NSM (*tph-1::mCherry*) for each given genotype, food level, and temperature (see Table S4). Bar in lower left panel indicates 10 μm.

Figure S5

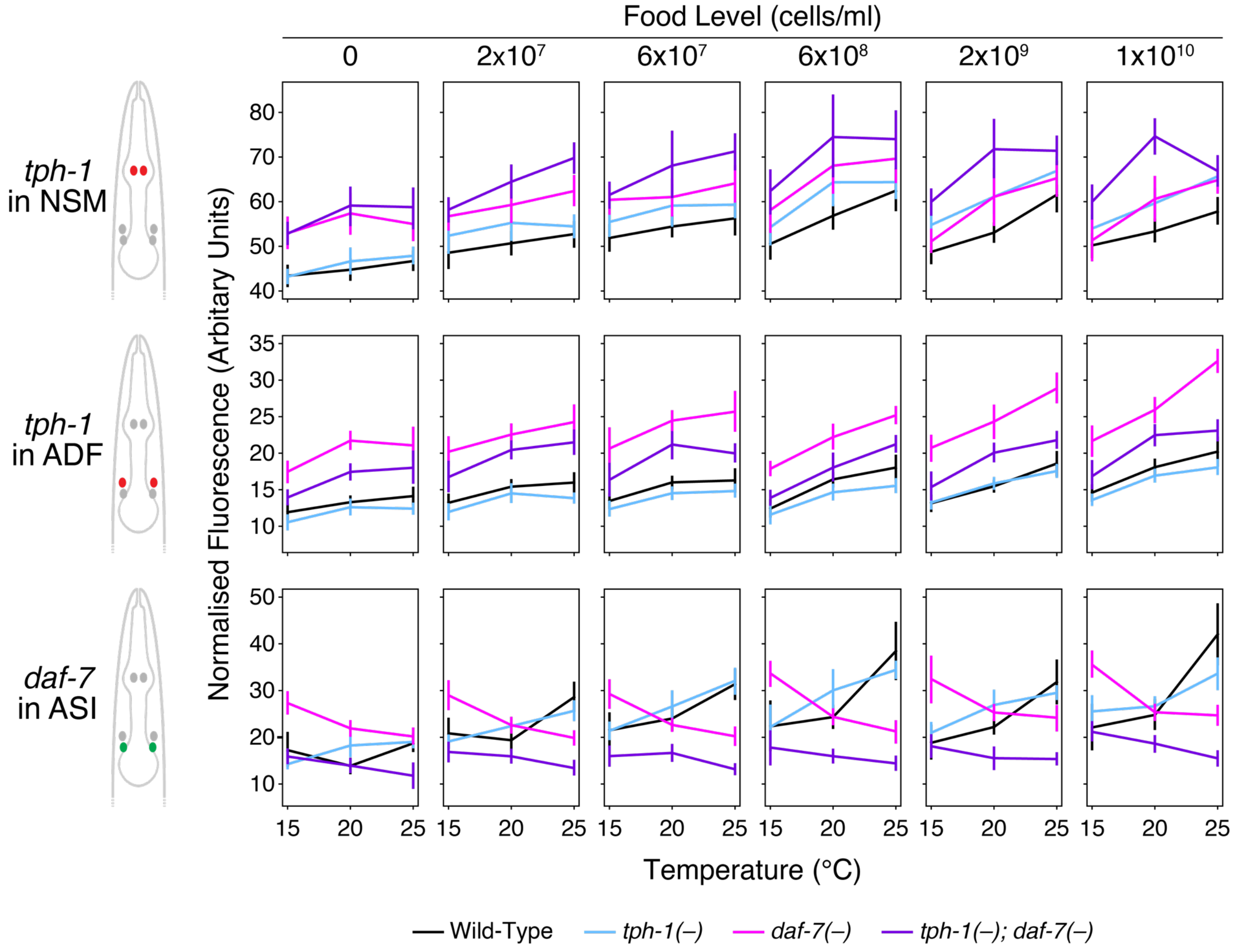

Figure S5. Effect of temperature on *tph-1* and *daf-7* expression as a function of food level. Related to Figure 5. Bayesian estimates for the normalized expression levels of *tph-1::mCherry* in NSM and ADF, and *daf-7::Venus* in ASI. Range of sample sizes for the 18 food and temperature conditions tested are as follows: wild-type n = 405 to 2958, *tph-1(-)* n = 70 to 313, *daf-7(-)* n = 90 to 191, *tph-1(-); daf-7(-)* n = 57 to 145 (see Table S4 for detailed sample sizes). Error bars denote 90% confidence intervals (see Table S4 for values).

Figure S6

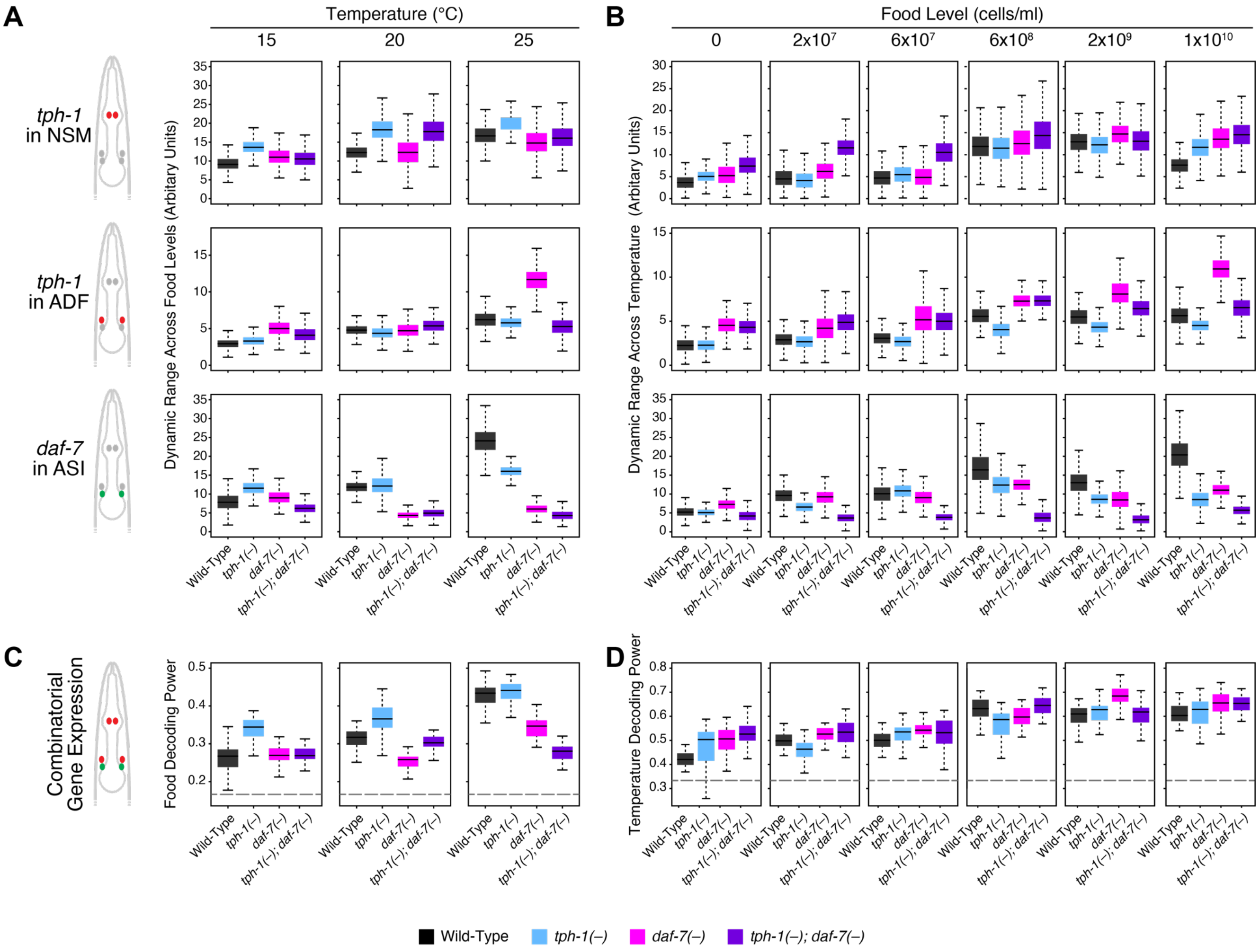

A
B
C
D
Temperature (°C)
15
20
25
Food Level (cells/ml)
0
2x10^7
6x10^7
6x10^8
2x10^9
1x10^10
tph-1 in NSM
tph-1 in ADF
daf-7 in ASI
Combinatorial Gene Expression
Dynamic Range Across Food Levels (Arbitary Units)
Dynamic Range Across Temperature (Arbitary Units)
Food Decoding Power
Temperature Decoding Power
Wild-Type
tph-1(–)
daf-7(–)
tph-1(–); daf-7(–)

Figure S6. Effects of genotype, temperature, and food on dynamic range and decoding power of *tph-1* and *daf-7* expression. Related to Figures 4 and 5. (A) Dynamic range of food-responsive *tph-1* expression in NSM and ADF, and *daf-7* expression in ASI at different temperatures. See Table S5 for quantification of overlap between distributions depicted in box plots. Diagrams on the left indicate the gene and cell. **(B)** Dynamic range of temperature-responsive *tph-1* expression in NSM and ADF, and *daf-7* expression in ASI at different food levels. **(C)** Food decoding power based on combinatorial expression of *tph-1* and *daf-7* in all three cells at each temperature. See Table S6 for quantification of overlap between distributions depicted in box plots. **(D)** Temperature decoding power based on combinatorial expression of *tph-1* and *daf-7* in all three cells at different food levels. Range of sample sizes for the 18 food and temperature conditions tested are as follows: wild-type n = 405 to 2958, *tph-1(-)* n = 70 to 313, *daf-7(-)* n = 90 to 191, *tph-1(-); daf-7(-)* n = 57 to 145 (see Table S4 for detailed sample sizes). Bayesian distributions in (A)-(D) are depicted by boxplots as described for Figure 1E. Details of the decoding accuracies are shown in Figure S7.

Figure S7

A

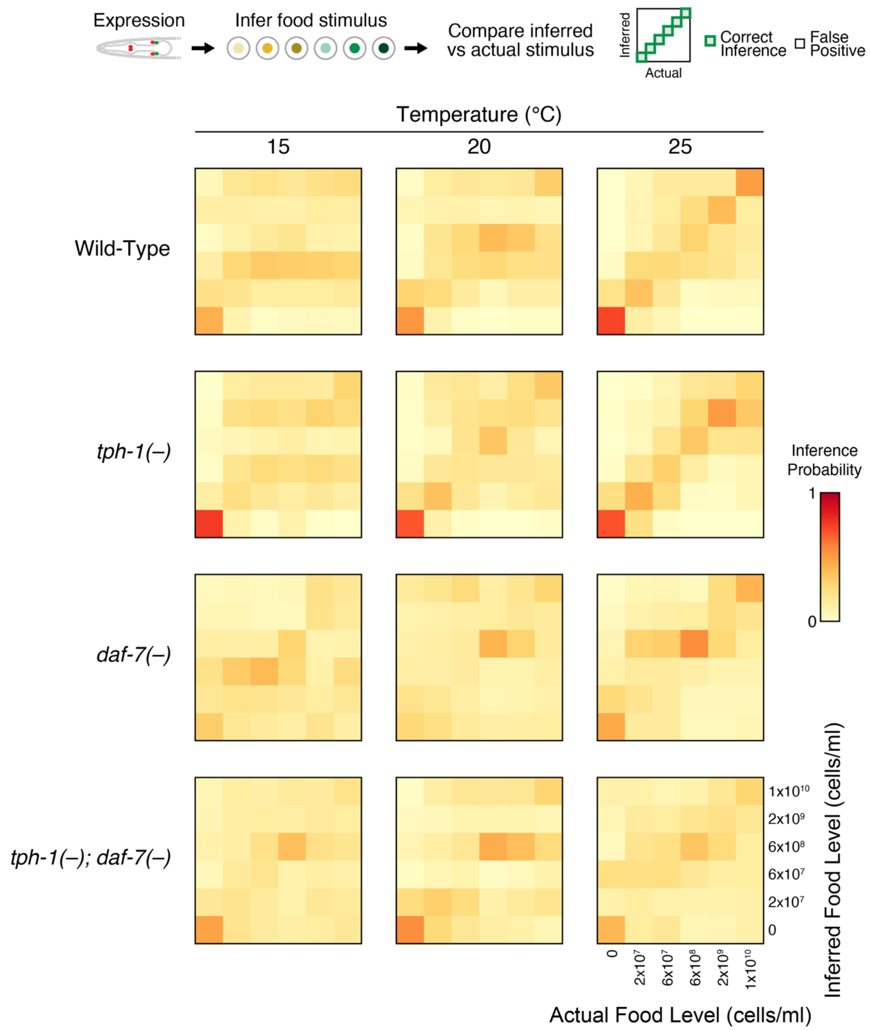
Expression
Infer food stimulus
Compare inferred vs actual stimulus
Inferred
Actual
Correct Inference
False Positive
Temperature (°C)
15
20
25
Wild-Type
tph-1(–)
daf-7(–)
tph-1(–); daf-7(–)
Inference Probability
1
0
1x10¹⁰
2x10⁹
6x10⁸
6x10⁷
2x10⁷
0
Inferred Food Level (cells/ml)
Actual Food Level (cells/ml)

B

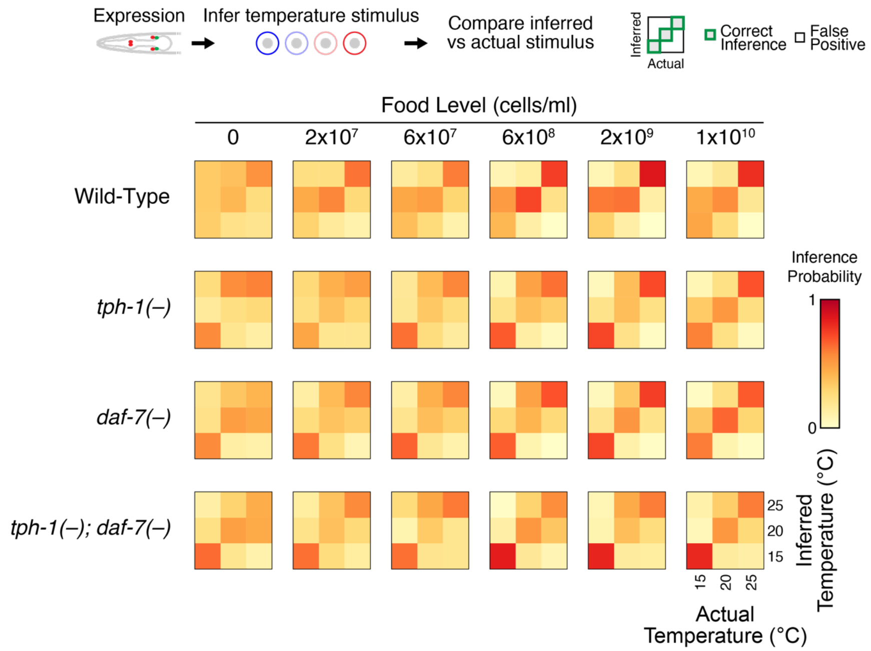
Expression
Infer temperature stimulus
Compare inferred vs actual stimulus
Inferred
Actual
Correct Inference
False Positive
Food Level (cells/ml)
0
2x10⁷
6x10⁷
6x10⁸
2x10⁹
1x10¹⁰
Wild-Type
tph-1(–)
daf-7(–)
tph-1(–); daf-7(–)
Inference Probability
1
0
25
20
15
Inferred Temperature (°C)
Actual Temperature (°C)

Figure S7. Accuracy of decoding food or temperature from gene expression. Related to Figures 4 and 5. (A) Top: summary of the food decoding process using gene expression. Bottom: confusion matrices depicting food decoding at different temperatures in different genotypes. **(B)** Top: summary of the temperature decoding process using gene expression. Bottom: confusion matrices depicting temperatures decoding at different food levels in different genotypes. Range of sample sizes for the 18 food and temperature conditions tested are as follows: wild-type n = 405 to 2958, *tph-1(-)* n = 70 to 313, *daf-7(-)* n = 90 to 191, *tph-1(-); daf-7(-)* n = 57 to 145 (see Table S4 for detailed sample sizes).

Figure S8

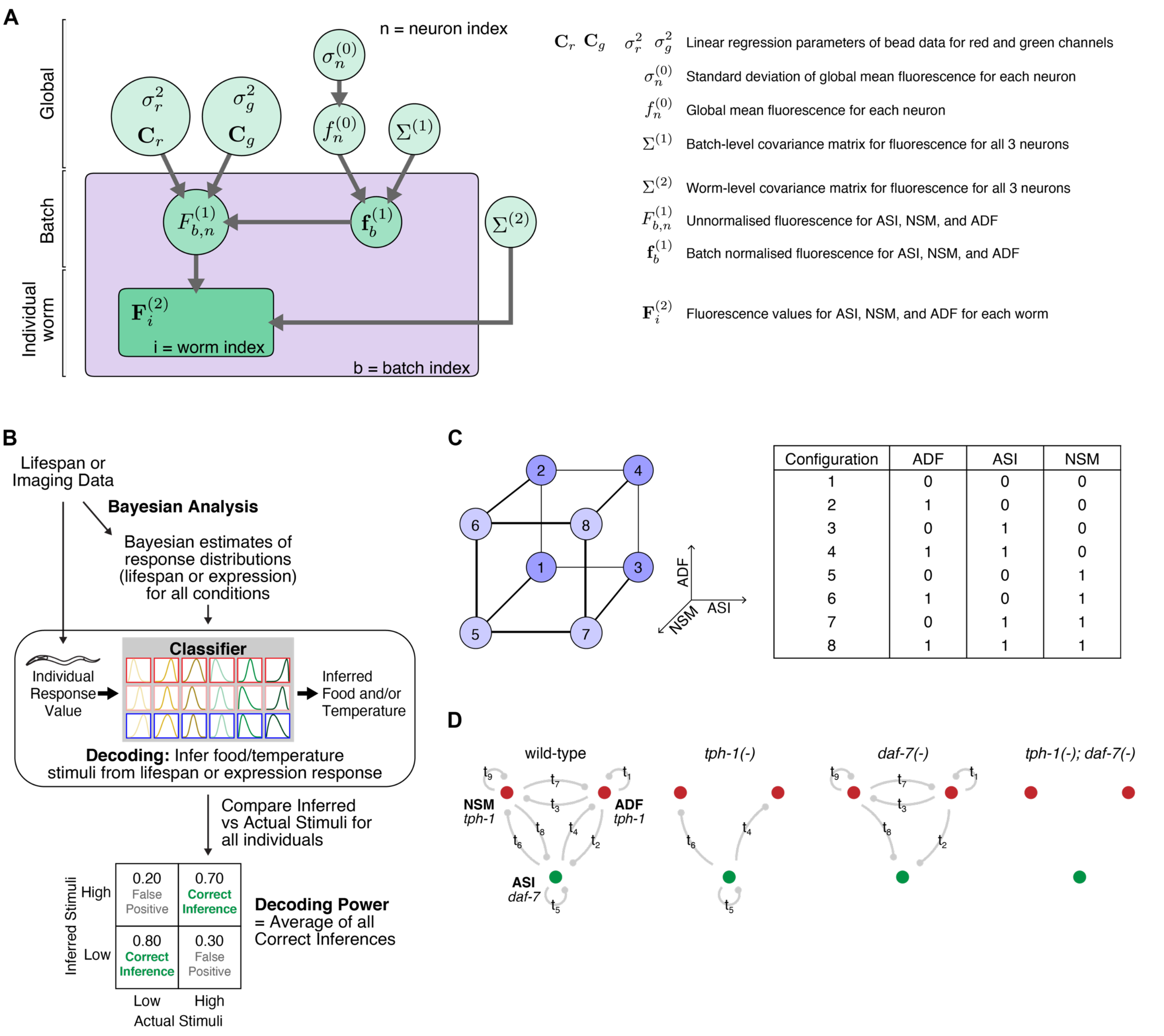

Configuration	ADF	ASI	NSM
1	0	0	0
2	1	0	0
3	0	1	0
4	1	1	0
5	0	0	1
6	1	0	1
7	0	1	1
8	1	1	1

Figure S8. Bayesian analysis and network modelling. Related to Figure 6 and STAR Methods. **(A)** Hierarchical Bayesian model for estimating the expression values of ASI, ADF, and NSM. **(B)** Schematic of the decoding analysis procedure. **(C)** Model representing possible states of ASI, ADF, and NSM and the transitions between these states (see Star Methods for details). **(D)** The network is modelled with terms (*t_1* to *t_9*) for each directed cell-cell regulation. Mutants were represented in the network models as the absence of edges emanating from the cell normally expressing the mutated gene.
